# Alkaline-Induced Degradation Pathways of β‑*O*‑4-Linked Vanillin Moieties Produced during Lignin Oxidation and the Effect of Na^+^‑Cyclic Polyether Complexes

**DOI:** 10.1021/acsomega.5c07658

**Published:** 2025-08-22

**Authors:** Yuki Hirano, Takashi Hosoya, Hisashi Miyafuji

**Affiliations:** Graduate School of Life and Environmental Sciences, 12897 Kyoto Prefectural University, Japan. 1-5 Shimogamo-hangi-cho, Sakyo-ku, Kyoto 606-8522, Japan

## Abstract

The production of vanillin (4-hydroxy-3-methoxybenzaldehyde) from lignin via alkaline aerobic oxidation offers a viable approach for synthesizing aromatic compounds from biomass. A principal route for vanillin formation involves the liberation of a vanillin molecule through nonoxidative, alkali-induced ether cleavage at the β-*O*-4 type nonphenolic vanillin end group. Our previous studies using veratraldehyde, a model for the vanillin end, showed that complex cations formed between crown ethers and Na^+^ enhanced vanillin release. In this study, to gain delve deeper into the mechanisms controlling the vanillin elimination, we used a vanillin end group model, 4-[2-(3-ethoxy-4-methoxy-phenyl)-2-hydroxy-1-(hydroxymethyl)­ethoxy]-3-methoxy-benzaldehyde, **VE**_**β**_. It was subjected to degradation in the presence of various complex cations under nonoxidative alkaline conditions (4.0 mol/L NaOH aq. at 120 °C), mirroring our previous experiments. Upon dissolution in alkaline solution, **VE**_**β**_ underwent rearrangement of the ether-linked vanillin residue to α- and γ-positions. The subsequent heating induced vanillin elimination, while side reactions such as polymerization also occurred, reducing vanillin selectivity. The presence of a complex cation between the crown ether, 15-crown-5, and Na^+^ improved the selectivity for vanillin production while mitigating the polymerization pathway. In contrast, other complex cations, though previously effective in promoting vanillin formation from native lignin, did not enhance the yield from **VE**_**β**_. These contrasting results suggest that in native lignin, additional vanillin production pathways originating from interunit linkages beyond the β-*O*-4 linkage may contribute to the overall product distribution.

## Graphical abstract

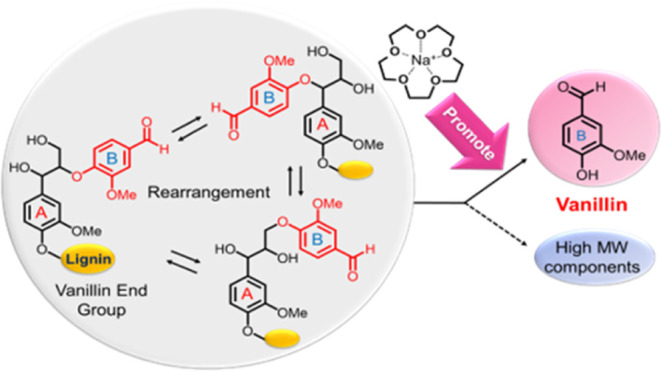

## Introduction

Lignin is an aromatic polymer that accounts for 15–35 wt % of lignocellulose and is expected as a renewable source for various aromatic chemicals critical to industry.^1^ Numerous depolymerization techniques have been explored to obtain low-molecular-weight (MW) aromatic compounds for chemical industry applications via lignin depolymerization.^2–9^ Among these approaches, oxidative degradation under alkaline conditions, known as alkaline aerobic oxidation, has proven effective in cleaving the primary interunit linkages in lignin, especially the ether bonds. This process facilitates the generation of low MW phenols via oxidative degradation of the C_3_ side-chains in lignin molecules.^3,10–19^ Additionally, this method is particularly promising for lignin conversion as it utilizes molecular oxygen—a nontoxic and widely available oxidant present in air.

We have been developing a method for producing vanillin (4-hydroxy-3-methoxybenzaldehyde) from native lignin, focusing particularly on wood flour, a byproduct abundantly generated during wood processing. Vanillin is an important compound in the chemical industry, used not only as a flavoring agent but also in pharmaceutical synthesis.^20–23^ Our recent studies have demonstrated that vanillin can be efficiently produced by treating softwood flour at 120 °C in a concentrated alkaline solution ([OH^–^] = ∼4.0 mol/L) containing bulky organic cations, in the presence of molecular oxygen.^17,24,25^ Notably, in a reaction medium containing crown ethers—specifically 18-crown-6 (1,4,7,10,13,16-hexaoxacyclooctadecane, **18C6**), 15-crown-5 (1,4,7,10,13-pentaoxacyclopentadecane, **15C5**), and 12-crown-4 (1,4,7,10-tetraoxacyclododecane, **12C4**) (Scheme 1)—in a 4.0 mol/L NaOH solution, vanillin yields of 15.2–16.1 wt % were achieved based on Klason lignin content of Japanese cedar (*Cryptomeria japonica*).^24^ This high yield is not observed in systems without crown ethers, nor in those containing 2,5,8,11,14-pentaoxapentadecane (tetraglyme, **TEG**) (Scheme 1), a linear analog of **15C5**, or 1,4-dioxane, which has a significantly smaller ring size. These results indicate that the complex cations formed by the crown ethers and Na^+^ play a catalytic role in vanillin production.^24^

**Scheme 1:**
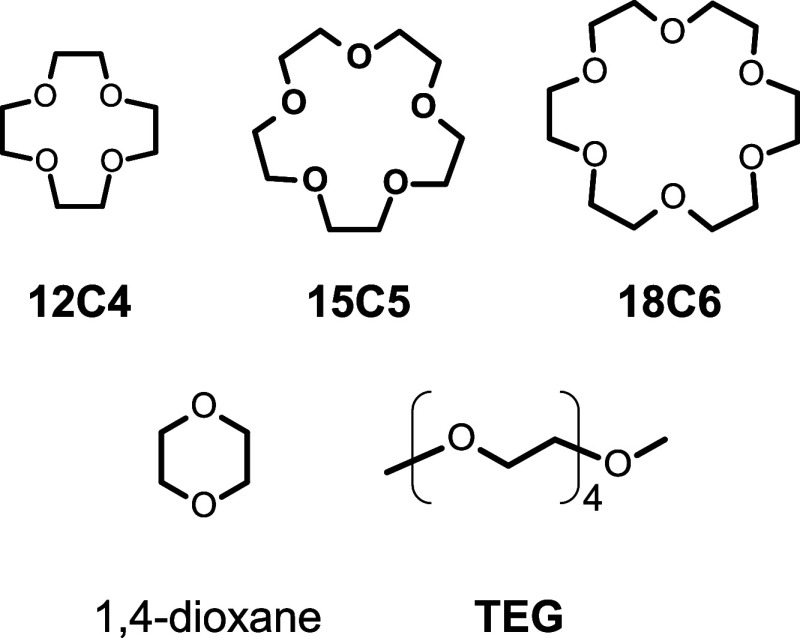
Crown Ethers and Other Ethers Employed as Additives in This Study

Our model experiments have also suggested an important vanillin formation pathway as outlined in Scheme 2. Specifically, in the aerobic oxidation of the intermediate units with β-*O*-4 structures, which account for approximately 50% of the interunit linkages in lignin, the glycerol end group is initially formed through the alkaline hydrolysis of the β-ether bonds. The side-chain of this end group is then oxidized and transformed into the vanillin end group.^26^ Subsequently, the vanillin molecule is released through the alkaline hydrolysis of the β-ether bond immediately preceding the vanillin residue. Additionally, as a simple model for the vanillin end group in this pathway, veratraldehyde (3,4-dimethoxybenzaldehyde) was used, as shown in Scheme 3, to investigate the effects of various organic cations on its conversion to vanillin under alkaline conditions. The results revealed that complex cations suppress the bimolecular disproportionation pathway, in which veratraldehyde is converted into the corresponding alcohol **I** and carboxylic acid **II** (Scheme 3). This suppression is attributed to the “cage effect”, wherein these cations surround the veratraldehyde molecule like a cage, thereby inhibiting molecular collisions. As a result, the competing vanillin formation pathway is suggested to become more favorable under these conditions.^24,25^

**Scheme 2:**
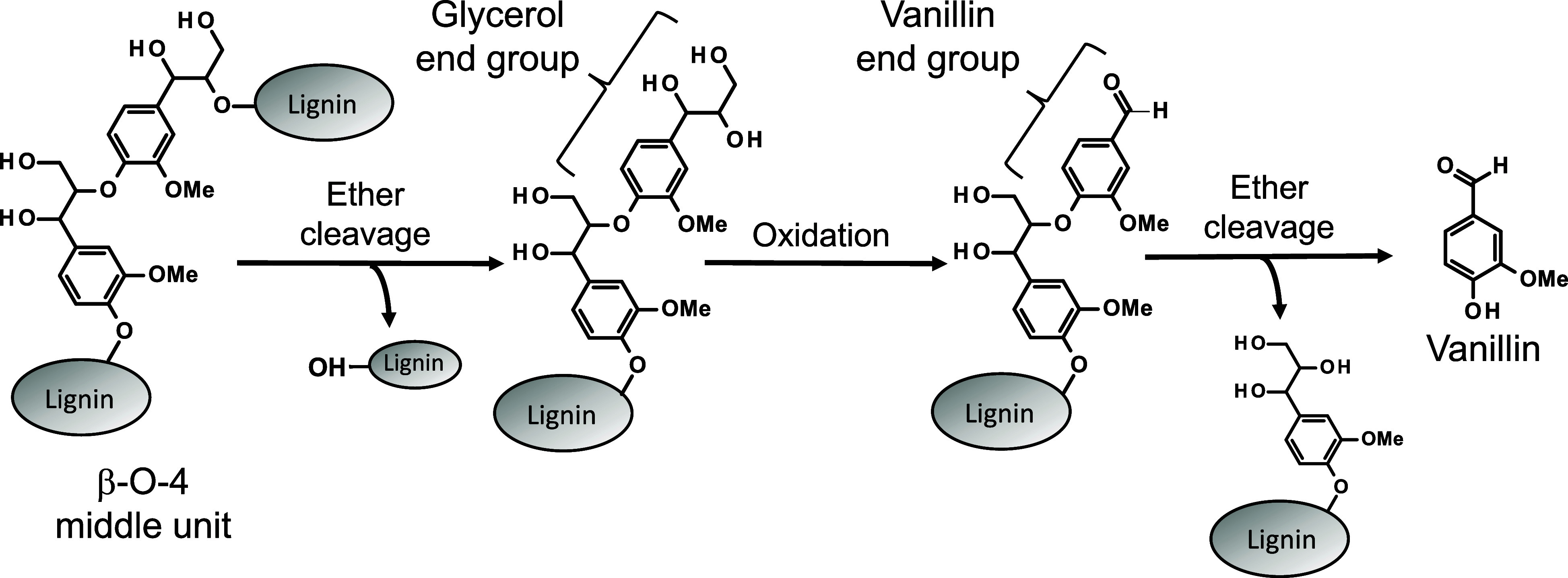
A Vanillin Production Pathway Proposed in Our Previous Studies^24,25,27,28^ from a β-*O*-4 Middle Unit of Lignin to Monomeric Vanillin During Alkaline Aerobic Oxidation^a^

a Adapted with permission from ref 25. Copyright 2020 Royal Society of Chemistry

**Scheme 3:**
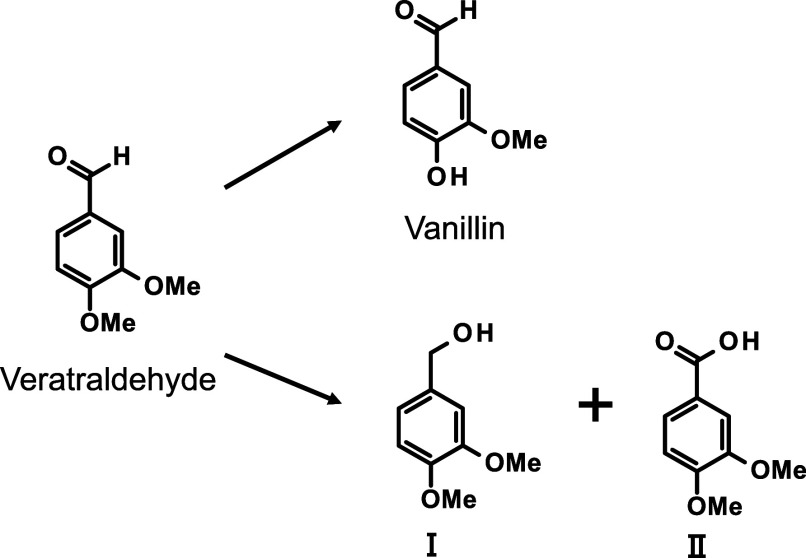
Vanillin-Producing and Disproportionation Pathways Proceeding from Veratraldehyde in Strong Aalkali^24–26^ ^a^

a Adated with permission from refs 25 and 26 . Copyright 2020 and 2022 Royal Society of Chemistry

The findings from our previous research indicate that the complex cations positively influence the final stage depicted in Scheme 2, specifically the elimination of vanillin from the vanillin end group. This elimination process is thought to be primarily facilitated by nonoxidative alkaline hydrolysis of ether bonds. The molecular mechanisms underlying this ether cleavage have been explored in the context of lignin depolymerization reactions during the production of chemical pulp. The cleavage of β-ethers, assisted by neighboring groups such as α-hydroxy groups, is a well-documented phenomenon.^29,30^ Contrary to the cleavage of β-*O*-4 type intermediate units in native lignin, the vanillin elimination reaction illustrated in Scheme 2 involves an aldehyde functional group in the side-chain of the leaving group. Although research into the cleavage mechanism of β-ethers under these specific conditions is scarce, the presence of an aldehyde group in the leaving group has been shown to (1) accelerate the rate of the β-ether cleavage and (2) facilitate reactions in which the leaving group migrates to other positions on the side chain, such as the α-position.^31–33^

The reaction conditions under which the β-ether cleavage mechanism was investigated in alkaline cooking for 2 manufacturing differ significantly from those used in our vanillin production process, particularly in terms of alkali concentration and reaction temperature. In our process, higher alkali concentrations and lower temperatures are employed compared to typical pulping conditions. Moreover, the model compounds used in the aforementioned prior studies,^31–33^ which possess an aldehyde group in the leaving group, are those with a C_2_ side-chain (2-(4-formyl-2-methoxyphenoxy)-1-(3,4-dimethoxyphenyl) ethanol), and no reaction behavior has been reported for model compounds with a C_3_ side-chain, which are structurally closer to those found in actual lignin. It is also worth noting that, while the degradation behavior of veratraldehyde—a model compound we previously used to represent the vanillin end group—has been studied under our specific vanillin production conditions, its chemical structure differs substantially from that of the vanillin end group formed during the degradation of native lignin.

Given the challenges outlined above, this paper focuses on a model compound, 4-[2-(3-ethoxy-4-methoxy-phenyl)-2-hydroxy-1-(hydroxymethyl)­ethoxy]-3-methoxy-benzaldehyde, **VE**_**β**_ (Scheme 4), which more accurately mimics the structure of the vanillin end group produced through the degradation of actual lignin. This study investigates its nonoxidative reaction behavior under alkaline conditions, with particular attention to not only the production of vanillin from the β-ether cleavage, but also the progression of the disproportionation reaction of the aldehyde group within **VE**_**β**_ and the rearrangement of the vanillin residue at the β-position. None of the above reactions of **VE**_**β**_ require an oxidizing agent. In light of this, we adopted reaction conditions under a nitrogen atmosphere as a first step toward fully elucidating the reaction mechanism, while keeping in mind that an oxidative environment is employed in the actual degradation of lignin. Furthermore, by comparison with erythro-veratryl glycerol-β-guaiacyl ether (**VG**) (Scheme 4), a β-*O*-4 type model compound lacking the aldehyde group in the leaving group, the influence of the aldehyde group’s presence on the reaction characteristics of the model compounds is thoroughly investigated. Notably, **VE**_**β**_ differs by featuring an ethoxy group in place of a methoxy group on one of its two benzene rings (A-ring), enhancing the accuracy in distinguishing between products derived from A- and B-rings. In the latter part of this paper, we also discuss the effects of complex cations derived from crown ethers (Scheme 1) on the degradation reaction of **VE**_**β**_, building on previous our findings.

**Scheme 4:**
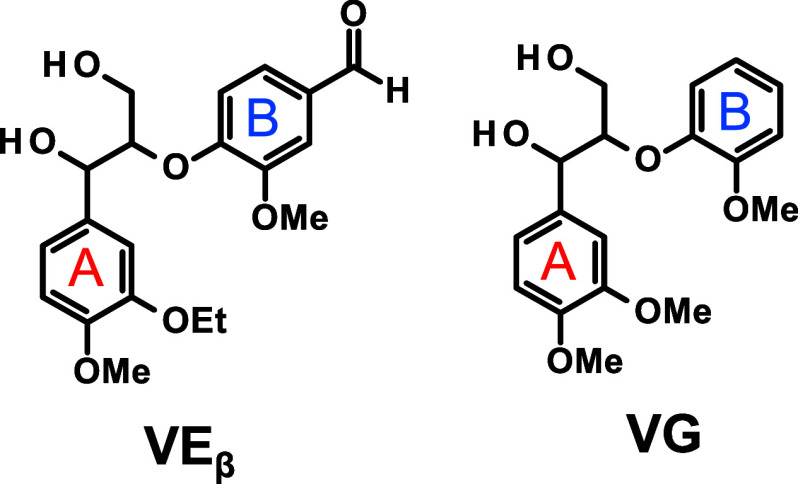
Lignin Model Compounds Employed in This Study^a^

a In these compounds, the two benzene rings are distinguished as the A- and B-rings.

This study aims to elucidate the reaction mechanism, particularly that proceeding from the vanillin end group, under the conditions we have employed in our previous work,^24,25^ and to investigate the influence of complex cations on this mechanism. The accumulation of fundamental insights obtained through this study is expected not only to contribute to addressing the challenges associated with existing vanillin production processes, but also to facilitate the future development of more cost-effective and environmentally friendly production technologies.

## Experimental Section

### Materials

For the alkaline aerobic oxidation experiments, **VE**_**β**_ and **VG** as shown in Scheme 4 were used as samples (The ratio of erythro to threo forms in **VE**_**β**_ and **VG** were 1:0.08 and 1:0.03, respectively). Additionally, as standards for the identification of degradation products, 1-(3-ethoxy-4-methoxy-phenyl)-2-[4-(hydroxymethyl)-2-methoxy-phenoxy] propane-1,3-diol **D**_**1**_ and 4-[2-(3-ethoxy-4-methoxy-phenyl)-2-hydroxy-1-(hydroxymethyl)­ethoxy]-3-methoxy-benzoic acid **D**_**2**_ were synthesized. Detailed synthesis methods and spectral data for these compounds are described separately in the Supporting Information under the synthesis of model compounds (see pages Figures S13–S20 in the Supporting Information).

**12C4**, **15C5**, **18C6**, 1,4-dioxane, and tetraglyme (**TEG**) (Scheme 1) were purchased from FUJIFILM Wako Pure Chemical Corporation. High-performance liquid chromatography (HPLC) grade acetonitrile and tetrahydrofuran were also obtained from the same company.

### Alkaline Degradation and Product Analysis

An ethanol solution of the lignin model compound (**VG** or **VE**_**β**_), adjusted to a precise concentration, was added to a 5.0 mL perfluoroalkoxy alkane (PFA) test tube, so that the weight of the introduced model compound was 3.0 mg (**VG**: 8.97 μmol, **VE**_**β**_: 7.97 μmol). After the solution in the test tube was removed under reduced pressure, the interior was replaced with nitrogen in a glovebox, and 2.0 mL of a 4.0 mol/L sodium hydroxide solution was added, followed by sealing the test tube with a silicone stopper. As shown in Figure 1, this test tube was placed inside a pressure tube (20.0 mL capacity) filled with 3.0 mL of distilled water, and then the pressure tube was tightly sealed. The reaction was initiated by immersing the pressure tube in an oil bath preheated to 120 °C and stirring for 4 h. Experiments were also conducted in a similar manner with the system containing additives such as **12C4**, **15C5**, **18C6**, 1,4-dioxane, **TEG** (2.0 mmol) added to the NaOH aq. These experiments were identical in terms of NaOH concentration and the amount of polyether additives to those in our previous study.^24^

**Figure 1:**
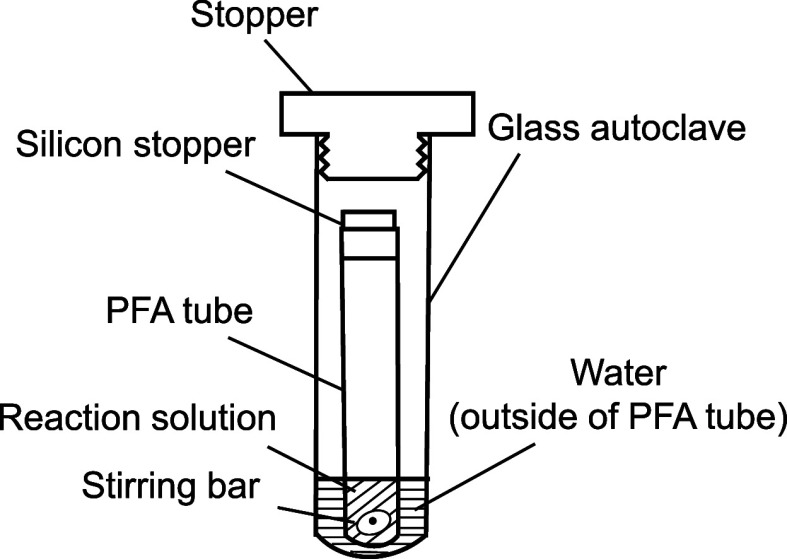
Experimental system employed in this study.

After cooling the pressure tube with water, the internal test tube was opened, and 1.0 mL of ethanol solution containing of a precisely weighed internal standard (*p*-hydroxybenzaldehyde, ∼1 mg) was added to the reaction mixture. Of the resulting solution, 20 μL was diluted with 980 μL of a 0.08 mol/L trifluoroacetic acid aqueous solution/acetonitrile solution = 9/1 (v/v), syringe-filtered through a 0.45 μm filter, and the obtained solution was used as a sample for HPLC analysis. Additionally, a sample preparation was carried out by simply dissolving **VE**_**β**_ in the NaOH aq. at room temperature, and this sample was designated as the 0 h reaction time sample.

For HPLC analysis, the system utilized was equipped with components manufactured by Shimadzu Corporation, including a pump (LC-20AD), a column oven (CTO-20AC), and a photodiode array detector (SPD-M40). The analytical conditions were as follows: Cadenza CD-C18 (Imtakt), column temperature 35 °C, flow rate 0.8 mL/min, with a mobile phase of 0.1% trifluoroacetic acid aqueous solution/acetonitrile solution (90/10 → 45/55 0–30 min, 45/55 → 0/100 30–35 min, 0/100 35–40 min, 0/100 → 45/55 40–45 min, 45/55 → 90/10 45–50 min). Product identification was achieved by comparing the retention times with those of commercially available authentic compounds. For compounds such as 1-(3-ethoxy-4-methoxy-phenyl) propane-1,2,3-triol, **ArG**, and 4-[3-(3-ethoxy-4-methoxy-phenyl)-2,3-dihydroxy-propoxy]-3-methoxy-benzaldehyde, **VE**_**γ**_, which were not commercially available, isolation was performed from the reaction mixture of **VE**_**β**_ using medium-pressure liquid chromatography (refer to the Supporting Information for detailed procedures), followed by acetylation with acetic anhydride/pyridine and structural analysis via ^1^H NMR spectroscopy. Quantification of products relied on comparing the peak areas of the internal standard to those of the products, with calibration curves for this quantification being established through HPLC analysis of the authentic compounds alongside the internal standard.

Due to the limited quantities isolated, calibration curves for **ArG** and **VE**_**γ**_ could not be generated. Consequently, for **ArG**, the calibration curve for veratryl glycerol (1-(3,4-dimethoxyphenyl)­propane-1,2,3-triol, **VGL**) (a 3-methoxy analog from a prior study) was utilized,^26^ and for **VE**_**γ**_, the calibration curve of **VE**_**β**_ was employed for quantification purposes. The validity of this substitution is supported by the structural similarities among the compounds, particularly in terms of molecular weight and functional groups that influence molecular properties (notably, **VE**_**β**_ and **VE**_**γ**_ share the same molecular weight).

The gel permeation chromatography (GPC) analysis of the reaction mixture was carried out according to the following procedure. After the reaction, the test tube, which was allowed to return to room temperature, was acidified by adding 10% hydrochloric acid, and then the solution was extracted with ethyl acetate. The obtained organic layer was washed once with brine, dried under reduced pressure, and then acetylated by adding 0.2 mL each of pyridine and acetic anhydride. The resulting solution was dried under reduced pressure and the residue was redissolved in 1.0 mL of ethanol. Of this solution, 20 μL was diluted with 980 μL of HPLC-grade tetrahydrofuran, syringe-filtered through a 0.45 μm filter, and used as a sample for GPC analysis. The GPC analysis was performed using the same analytical equipment as the HPLC analysis. GPC columns, KF-802 and KF-801 (Shodex) connected in this order, were used, with a column temperature of 35 °C, a flow rate of 1.0 mL/min, and tetrahydrofuran as the eluent.

## Results and Discussion

### Degradation Pathways of VE_β_

#### Vanillin-Producing Pathways

Figure 2A presents the HPLC chromatogram of **VE**_**β**_ at a reaction time of 0 h (the sample was only dissolved in 4.0 mol/L NaOH aq. at room temperature). This chromatogram exhibited multiple peaks between retention times of 21.0 to 24.0 min, in addition to the peak of **VE**_**β**_ at a retention time of 20.3 min. These results indicate that simply dissolving **VE**_**β**_ in an alkaline solution promotes its degradation. We isolated some of the compounds corresponding to these peaks using medium pressure liquid chromatography. Analysis of the isolated compounds by ^1^H NMR suggested that the compound with a retention time of 22.3 min was **VE**_**γ**_ (erythro form), in which the vanillin residue had migrated to the γ-position (for details on the isolation method and spectra, refer to the Supporting Information, pages Figures S2–S7). Although structural analysis by ^1^H NMR has not been conducted for the compounds corresponding to the closely eluted twin peaks at retention times of 21.2 to 21.4 min, one of these peaks may be attributed to **VE**_**α**_, in which the vanillin residue has migrated to the α-position. The quantitative results for the HPLC-detected compounds are shown in entry 1 in Table 1. Simply dissolving **VE**_**β**_ in the alkaline solution resulted in 24% degradation of **VE**_**β**_, with **VE**_**γ**_ being formed in a yield of 71 mol % based on the degraded **VE**_**β**_. Assuming one of the peaks at 21.2 to 21.4 min to be the rearranged product to the α-position, **VE**_**α**_, the total yield of these rearranged products would be expected to increase further. These results indicate that most of the constituents in the reaction mixture at a reaction time of 0 h can be accounted for by unreacted **VE**_**β**_ and rearranged products such as **VE**_**γ**_. Additionally, vanillin formation was minimal at 0 h [yield: 1 mol %], strongly suggesting that the alkaline hydrolysis of the ether bonds progresses very slowly at room temperature.

**Figure 2:**
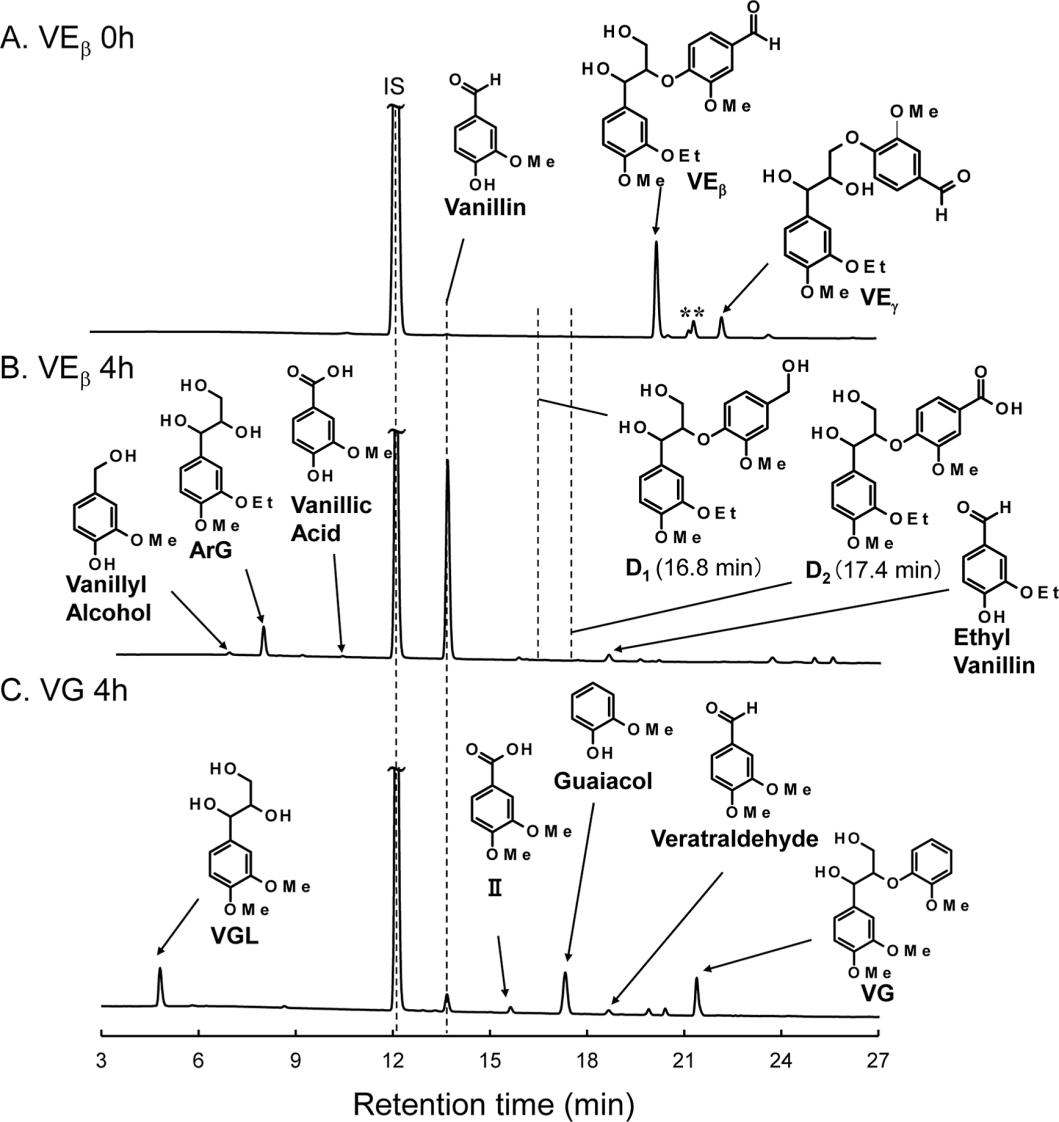
HPLC chromatograms of reaction mixtures obtained from the degradation of **VE**_**β**_ for 0 h (A) and 4 h (B), and **VG** for 4 h (C) in 4.0 mol/L NaOH aq. at 120 °C under N_2_. Detection wavelength: UV_280 nm._ The sample for 0 h was prepared by only dissolving **VE**_**β**_ in the NaOH aq. at room temperature (see Experimental Section). The identification of **VE**_**γ**_ and **ArG** was performed by ^1^ H NMR analysis of the isolated compounds (see Figures S1–S3). The retention times of **D**_**1**_ and **D**_**2**_ were indicated with dotted lines although these compounds were not detected in the reaction mixture. IS: Internal standard. The peaks marked with an asterisk (*) have not been identified, but it is likely that one of these peaks correspond to **VE**_**α**_.

**Table 1:**
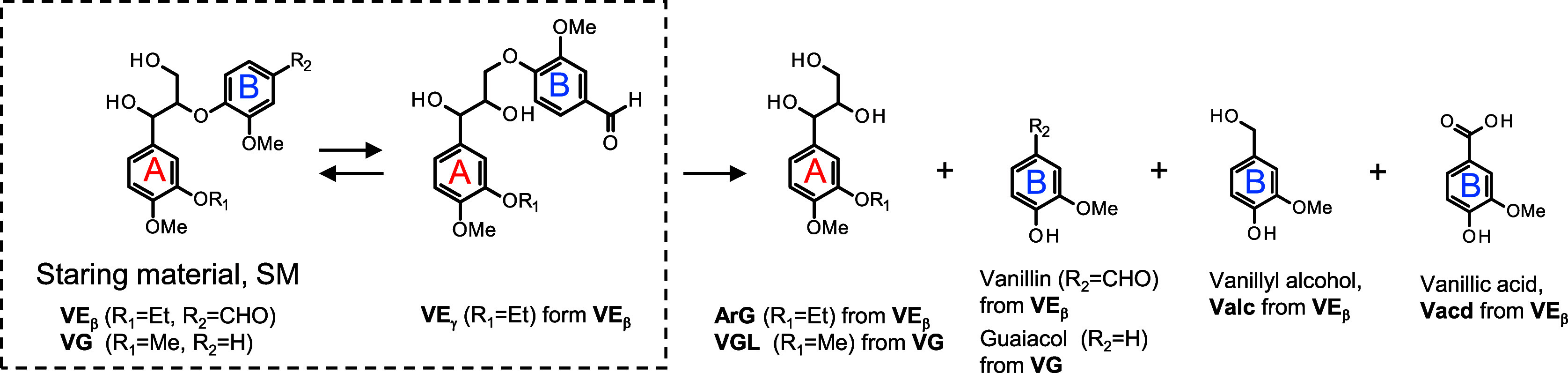
Product Yields and Recovery of the Starting Material after the Alkaline Degradation of **VE**_**β**_ and **VG** in 4.0 mol/L NaOH aq. at 120 °C under N_2_ ^i^

entry	SM^a^	medium	RT^b^ (h)	recovery (%)	product yield (mol %)^c^				
					**VE_γ_**	**ArG** or **VGL**	vanillin or guaiacol	Valc^d^	Vacd^e^
1	**VE**_**β**_	NaOH aq	0	76	17	ND^f^	1	ND	ND
2	**VE**_**β**_	NaOH aq	4	0.5^g^ [0.5, 0.4]^g^	ND	47 [47,47]	46 [45,46]	9 [8,10]	1 [1,1]
3	**VE**_**β**_	NaOH aq + **18C6**	4	ND	ND	48 [47,49]	34 [33,34]	4 [3,4]	ND
4	**VE**_**β**_	NaOH aq +**15C5**	4	ND	ND	67 [67,67]	63 [63,63]	9 [8,9]	2 [1,2]
5	**VE**_**β**_	NaOH aq + **12C4**	4	ND	ND	43 [42,43]	32 [32,32]	3 [2,3]	ND
6	**VE**_**β**_	NaOH aq + **TEG**	4	1	ND	42	33	4	ND
7	**VE**_**β**_	NaOH aq + 1,4-dioxiane	4	0.9 [0.6,1.2]	ND	49 [47,50]	47 [46,47]	9 [6,11]	1 [1,0.5]
8	**VG**	NaOH aq	4	27		44 (60)^h^	64 (88)		

a In This table, the product yields from the degradation experiments of **VE**_**β**_ and **VG** are grouped by category to allow direct comparison. For example, the yields of vanillin from **VE**_**β**_ and guaiacol From **VG** are presented in the same cell, as they are both derived from the B-ring. A conventionally formatted version of this table, in which the product yields are not grouped in this manner, is provided as Table S1 in the Supporting Information.

b SM: Starting material.

c RT: Reaction time.

d Product yield is based on the initial molar amount of the starting material.

e Valc: Vanillyl alcohol.

f Vacd: Vanillic acid.

g ND: not detected.

h For the items accompanied by brackets, the same experiment was conducted twice. The values inside the brackets indicate the yields obtained in each experiment, while the value outside the brackets represents the average yield from the two experiments.

i The number in the parentheses shows yields (mol %) of **VGL** or guaiacol on the basis of the degraded **VG**.

We next conducted a degradation experiment by heating a solution of **VE**_**β**_ in NaOH aq. at 120 °C for 4 h under nitrogen. As shown in the HPLC chromatogram in Figure 2B and the quantified values of the compounds detected by HPLC listed in entry 2 in Table 1, **VE**_**β**_ was completely degraded after 4 h of heating, resulting in the formation of **ArG** (retention time: 8.1 min) and vanillin (retention time: 13.9 min) at yields of 47 mol % and 46 mol %, respectively. None of the rearranged products, such as **VE**_**γ**_, which were detected at a reaction time of 0 h, were observed at this time point. These results indicate that the ether bonds in compounds such as **VE**_**β**_ and **VE**_**γ**_ were degraded at 0 h, leading to the release of vanillin. In this alkaline degradation experiment, ethylvanillin (3-ethoxy-4-hydroxybenzaldehyde) (retention time: 18.6 min) was formed at a yield of 2 mol % (not listed in Table 1). This compound was likely produced by the oxidation of the side-chain of **ArG** by residual O_2_ in the system.^26^ However, due to its minor quantity, further discussion is omitted in this paper.

Based on the above findings, a pathway for vanillin formation from **VE**_**β**_ was proposed, as illustrated in Scheme 5. This pathway suggests that the rearrangement of the vanillin residue in **VE**_**β**_ occurs via the formation of acetal intermediates **1** and **2**, initiated by the nucleophilic attack of the α- or γ-oxyanion at the 4-position of the B-ring upon simple dissolution of the compound in the alkaline solution. While **VE**_**γ**_ is the only rearranged product detected and successfully characterized by ^1^H NMR in this study, it is inferred that the formation of **VE**_**α**_—entailing the rearrangement of the vanillin residue to the α-position—is also underway, as mentioned above. Similar rearrangement reactions have been observed in compounds analogous to **VE**_**β**_.^32^ Additionally, the quantities of **VE**_**β**_ and **VE**_**γ**_ remained unchanged even after the alkaline solution of **VE**_**β**_ was left at room temperature for 30 min. This observation indicates that **VE**_**β**_ rapidly forms an equilibrium mixture of various rearranged compounds, presumably including **VE**_**α**_, upon dissolution in alkaline solution. We also conducted NMR analyses of **VE**_**β**_ in NaOD/D_2_O solution; however, intermediates **1** and **2** were not detected. This is presumably because these intermediates, which have lost their aromatic ring structures, are unstable and thus exist at extremely low concentrations in solution. Nevertheless, it should be emphasized that, without assuming the formation of these intermediates, the experimentally observed transfer of the vanillin residue from **VE**_**β**_ cannot be rationally explained.

**Scheme 5:**
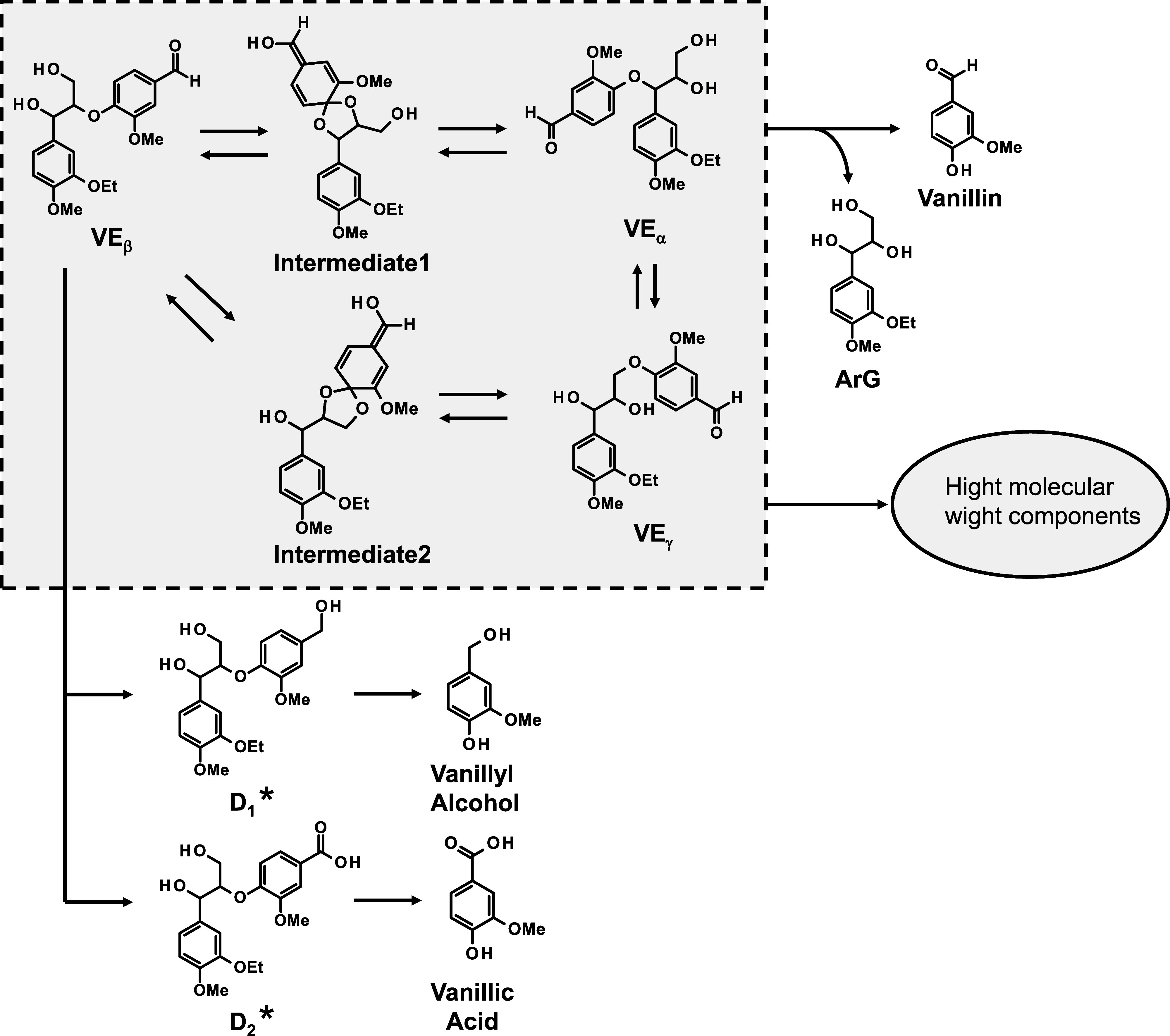
Reaction Pathways in the Alkaline Degradation of **VE**_**β**_

*For **D**_**1**_ and **D**_**2**_, alcohols and carboxylic acids akin to these compounds can also be generated from the other rearranged substances such as **VE**_**γ**_ and **VE**_**α.**_

Upon heating, the equilibrium mixture of **VE**_**β**_, **VE**_**α**_, and/or **VE**_**γ**_ undergoes ether bond cleavage, resulting in the elimination of vanillin. Two proposed mechanisms for this reaction include the S_N_icB mechanism, in which an alkoxide ion attacks the adjacent carbon, and the S_N_Ar mechanism, characterized by the nucleophilic attack of OH^–^ on the aromatic carbon (see Scheme S1 in the Supporting Information).^31,34,35^ The elucidation of these mechanisms is beyond the primary scope of this study. We present only a preliminary discussion regarding the possible reaction pathways, which is provided in the Supporting Information along with the corresponding schemes (see page S8 in the Supporting Information).

Our previous studies have reported that under the degradation conditions employed in this study (4.0 mol/L NaOH aq./120 °C/N_2_), a disproportionation reaction of veratraldehyde, a model compound with a highly simplified vanillin end group, proceeds, resulting in the formation of corresponding alcohol **I** and carboxylic acid **II** (Scheme 3).^26^ This disproportionation pathway was more favorable than the competing vanillin formation reaction under the above reaction conditions.^25^ Applying this insight into **VE**_**β**_, it is expected that a similar disproportionation reaction proceeds in **VE**_**β**_, resulting in the formation of disproportionated products **D**_**1**_ and **D**_**2**_ from the aldehyde group of the vanillin residue on the B-ring. To assess the contribution of the disproportionation pathway in the degradation of **VE**_**β**_, **D**_**1**_ and **D**_**2**_ were synthesized and analyzed by HPLC (Figure 2B). Although neither compound was clearly detected after 4 h of reaction, small amounts of vanillyl alcohol and vanillic acid—likely formed from the ether bond cleavage of **D**_**1**_ and **D**_**2**_—were observed, as summarized in entry 2 in Table 2. Trace levels of **D**_**1**_ and **D**_**2**_ were detectable at 2 h, along with minor unidentified peaks (see Figure S4 in the Supporting Information). These results suggest that while a disproportionation pathway involving the vanillin residue does occur, it is relatively minor in **VE**_**β**_ because the vanillin formation proceeds significantly faster than the competing disproportionation reaction under the present conditions (Scheme 5). A more in-depth discussion is presented in the Supporting Information (see pages S9 and 10 in the Supporting Information).

**Table 2:** Effects of Several Polyether Additives on the Degradation Behavior of **VE**_**β**_ Observed in This Study and on the Vanillin Yield during the Aerobic Oxidation of Japanese Cedar (*Cryptomeria japonica*) Wood Flour Reported in Our Previous Study.^24^ ^d^

	VE_β_ ^a^			wood^a^
	vanillin	vanillyl alcohol and vanillic acid	High-MW product^b^	vanillin
**18C6**	–^c^	–	+	+
**15C5**	+	±	–	+
**12C4**	–	–	+	+
**TEG**	–	–	+	±
1,4-dioxane	–	–	+	±

a Adapted in Part with Permission from ref 24. Copyright 2022 Taylor & Francis.

b **VE**_**β**_ data were obtained under a nitrogen atmosphere in this study, whereas softwood flour data were obtained under aerobic conditions in a previous study^24^.

c The presented effects were observed under conditions with 4.0 mol/L NaOH aq. at 120 °C, and in the presence of the additives equivalent to 1/4 molar of OH^–^.

d Meaning of the symbols: Increased (+), almost equal (±), and reduced (−) yield or production compared to the results in the absence of the additives.

#### Pathways Generating High-Molecular-Weight Compounds

The yield of vanillin after 4 h reaction of **VE**_**β**_ was 46 mol % (entry 2 in Table 1), which is less than half of the theoretical yield. Coupled with the fact that vanillin is a stable molecule under the experimental conditions of this paper,^25^ it is strongly suggested that even if vanillin end groups are formed within lignin molecules, as illustrated in Scheme 2, the elimination of vanillin does not proceed quantitatively. In other words, this implies that a considerable portion of the vanillin end formed through the various stages of the reaction pathway in Scheme 2 is rendered wasteful without giving vanillin as the final product. Hence, elucidating the reasons why vanillin elimination from **VE**_**β**_ is not quantitative is of utmost importance for improving the productivity of vanillin in the process.

Based on these considerations, we synthesized **VG**, a nonphenolic model compound lacking an aldehyde group in the leaving group, and compared its degradation behavior under 4.0 mol/L NaOH aq./120 °C/N_2_ with that of **VE**_**β**_. As shown in the HPLC chromatogram of the reaction mixture obtained from **VG** in Figure 2C, in addition to unreacted **VG** (21.3 min), several degradation products were detected: **VGL** (4.8 min), vanillin (13.6 min), carboxylic acid **II** (15.6 min), guaiacol (2-methoxyphenol) (17.3 min), and veratraldehyde (3,4-dimethoxybenzaldehyde) (18.6 min), along with some unidentified peaks at 19.9 and 20.4 min. Aromatic aldehydes and related substances that may have formed due to the residual oxygen, etc., will not be discussed further. Interestingly, as shown in entry 8 in Table 1, the yield of guaiacol, which is produced via the β-ether cleavage of **VG**, was nearly quantitative at 88 mol % based on the degraded **VG**.

The alkaline hydrolysis of the β-ether in **VG** is considered to proceed via the S_N_icB mechanism, as reported in many studies, involving the participation of an adjacent α-position oxyanion (see also Scheme S1 in the Supporting Information and discussion there for detailed explanation of this mechanism). In this case, rearrangement reactions of the guaiacyl group (B-ring) shown in Scheme 5 cannot occur. Thus, the difference in the yields of the ether-cleavage products (guaiacol and vanillin) between **VG** and **VE**_**β**_ is most likely due to the formation of the equilibrium mixtures shown in Scheme 5, specifically existing in the case of **VE**_**β**_. It should be noted that the yields of another set of degradation products, **VGL** and **ArG**, resulting from alkaline degradation, were not quantitative for either compound (entry 2 and 8 in Table 1). Although the cause of this is currently unclear, but there are studies mentioning reactions that could proceed through the epoxy intermediate, which is a precursor in the S_N_icB mechanism for these arylglycerols.^34^

To investigate the fate of the products from the degradation of **VE**_**β**_ that were not converted into vanillin and **ArG**, the reaction mixture obtained from **VE**_**β**_ was acetylated and then analyzed with GPC. As shown in Figure 3A, peaks corresponding to the acetylated derivatives of **ArG** (MW = 368.4) and vanillin (MW = 196.2), which are major products from **VE**_**β**_, appeared at elution times of 14.8 and 16.8 min, respectively. In addition, products with larger MWs than these were identified between elution times of 11.5 to 14.5 min (these products are hereafter referred to as “high MW components” in this paper). Subsequently, GPC analysis of the degradation products derived from **VG**, prepared under the same conditions, was conducted. As shown in Figure 3E, peaks corresponding to the acetylated derivatives of **VGL** and unreacted **VG** (MWs = 356.4 and 418.4, respectively) were detected at an elution time of 14.7 min, but the high MW components that appeared between 11.5 to 14.5 min in the case of **VE**_**β**_ were almost not detected in the case of **VG**.

**Figure 3:**
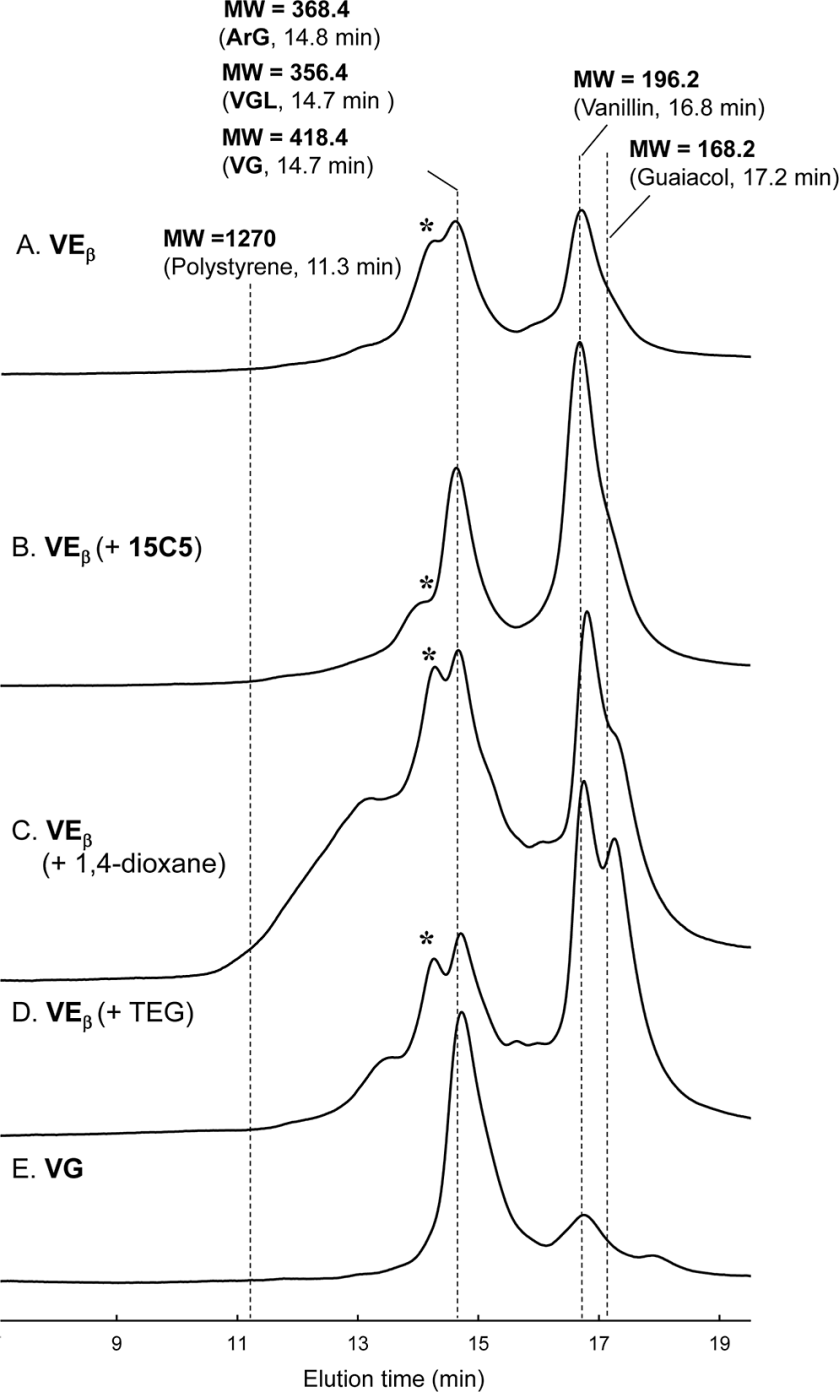
GPC Chromatograms of the acetylated reaction mixtures derived from the degradation of **VE**_**β**_ (A), **VE**_**β**_ in the presence of 1**5C5** (B), 1,4-dioxane (C), **TEG** (D), and **VG** (E) in 4.0 mol/L NaOH aq. at 120 °C for 4 h under N_2_. Detection wavelength: UV_280 nm_. The molecular weights of several acetylated authentic compounds were provided along with their elution times. In the chromatogram of sample E, a peak derived from acetylated guaiacol—a primary product from **VG**—was not observed, likely due to the volatilization of the compound during the sample preparation process. Peaks at 14.5 min, which are mentioned in the text, are indicated with an asterisk.

Combining the results from the GPC analysis with the differences in the yields of guaiacol from **VG** and vanillin from **VE**_**β**_, it can be concluded that, instead of quantitatively producing vanillin as a result of the ether bond cleavage from **VE**_**β**_, the high MW components are formed. Expanding on this idea, it is reasonable to attribute the primary reason for the nonquantitative production of vanillin from **VE**_**β**_ to the competition between the elimination reaction of vanillin from the equilibrium mixtures and the formation of the high MW components. The most significant mechanistic difference in the degradation of **VE**_**β**_ and **VG** is the formation of the equilibrium mixtures specific to **VE**_**β**_, as shown in Scheme 5. It is therefore considered that the high MW components generated from **VE**_**β**_ originate from one of the components in this equilibrium mixture. The specific chemical nature and formation mechanism of the high MW components are currently unclear. However, it is certain that these high MW components have significantly lower vanillin production capability than the dimeric compounds with ether bonds, such as **VE**_**β**_ or **VE**_**γ**_.

### Effects of Complex Cations

Our previous research showed that the addition of crown ethers (**12C4**, **15C5**, **18C6**) to the reaction system—or more precisely, the presence of complex cations formed between the crown ethers and Na^+^—led to an increase in the yield of vanillin from native lignin in alkaline oxidative degradation of softwood flour.^24^ As detailed earlier, one reason for this effect, as far as the results of simple model experiments with veratraldehyde are concerned, is the improved selectivity in the elimination of vanillin molecules from the vanillin end groups as shown in Scheme 2. At present, it is not clear whether complex cations exert a similar effect on the elimination of vanillin from **VE**_**β**_, which is a more realistic model of the vanillin end group. To this end, this section reports the results of degradation experiments of **VE**_**β**_ in the presence of complex cations and examines their effects on the reaction pathway outlined in Scheme 5.

#### Vanillin Production from **VE**_**β**_ in the Presence of **15C5**

We conducted degradation experiments of **VE**_**β**_ under the same conditions as mentioned above (4.0 mol/L NaOH aq./120 °C/4 h/N_2_), with the addition of **15C5**. Among the crown ethers used in this study (Scheme 1), **15C5** is the crown ether that best fits the size of Na^+^.^36,37^ As shown in entry 4 in Table 1, the addition of **15C5** resulted in qualitatively the same major products from **VE**_**β**_ as without addition, with vanillin and **ArG** being produced alongside the complete degradation of the starting material. On the quantitative side, however, the yield of vanillin increased significantly from 46 to 63 mol % with the addition of **15C5**. Furthermore, GPC analysis of the reaction mixture obtained with the addition of **15C5** revealed a reduction in the high MW components between elution times of 11.5 and 14.5 min compared to the system without addition (Figure 3A,B). In particular, the intensity of the peak corresponding to components with MWs slightly higher than that of **ArG** (near elution time 14.5 min, see peaks with an asterisk in Figure 3) was significantly reduced in the system with the addition of **15C5**.

The aforementioned results can be interpreted as the effect of the complex cation formed between **15C5** and Na^+^ tilting the relative rates of the opposing reactions, the formation of high MW components and the vanillin formation reaction, in favor of vanillin production. To further test this idea, **TEG** (Scheme 1), a noncyclic analogue of **15C5**, was added to examine its effect on the reaction behavior during the 4 h alkaline degradation of **VE**_**β**_. **TEG**, compared to **15C5**, has a significantly lower capacity for metal cation inclusion,^38–40^ which had been shown to have little positive effect on the yield of vanillin in the alkaline oxidative degradation of native softwood lignin.^24^ As shown in entrys 2 and 6 in Table 1, the addition of **TEG**, in contrast to the addition of **15C5**, resulted in a decrease in the yield of vanillin from 46 mol % to 33 mol % compared to the pure NaOH system. The GPC chromatogram of the reaction mixture obtained with **TEG** addition, shown in Figure 3D, observed an increase in the peaks of high MW components between elution times 12.5–14.5 min compared to the system without addition. These results suggest that in the **TEG**-added system, the polymerization pathway in Scheme 5 is favored over the vanillin production pathway, indicating an effect of **TEG** addition opposite to that of **15C5**. Notably, in the **TEG**-added system, the intensity of the peaks at 16.8 min (same as the elusion time of vanillin) and 17.4 min (also same as that of guaiacol) increased. However, since the yield of vanillin decreased in the **TEG**-added system (entry 6 in Table 1), this result suggests the presence of low MW products other than vanillin with increased yield in the **TEG**-added system. Indeed, the HPLC chromatogram of the **TEG**-added reaction solution detected a relatively large unidentified peak around 23.7 min (data not shown), which may explain the increase in the peak at 17.4 min observed in the GPC analysis.

Subsequently, we investigated the effects of adding 1,4-dioxane (Scheme 1), which is unlikely to form complex cations with Na^+^ due to its small ring size. As can be seen from the comparison between entries 2 and 7 in Table 1, the addition of dioxane resulted in a vanillin yield from **VE**_**β**_ nearly the same as that obtained with the NaOH alone system. Similar to the system with the **TEG** addition, an increase in high MW components between elution times 12.5–14.5 min was observed in the dioxane-added system (Figure 3C). Although the trend of increase in high MW components in the dioxane-added system was slightly stronger than in the **TEG**-added system, the shape of the GPC chromatogram in the high MW region before 15 min was quite similar in both systems. These results suggest that the effects of adding 1,4-dioxane and **TEG** on the degradation behavior of **VE**_**β**_ are similar in terms of the impact on vanillin yield and the formation of high MW components. A common feature of **TEG** and 1,4-dioxane is that both are ethers with lower polarity compared to water, and the similar effects of adding **TEG** and 1,4-dioxane are naturally attributed to such basic solvent properties. It is believed that further elucidation of the detailed reactions of high MW product formation will enable more concrete discussion on this solvent effect.

From the above discussion, we concluded that the increase in vanillin yield from **VE**_**β**_ and the suppression of the high MW component formation observed with the addition of **15C5** are related to the high inclusion ability of **15C5** toward Na^+^. Although **15C5** is an ether compound and, as such, should possess the potential to inhibit vanillin production similar to **TEG** and 1,4-dioxane, it is likely that the complex cation formed between **15C5** and Na^+^ exerts an effect strong enough to counteract this inhibitory effect. In our previous study examining vanillin production from veratraldehyde, it was considered that organic cations such as complex cations exert a cage effect on the reactants, thereby reducing the frequency of molecular collisions between them.^24^ Applying this concept to the current study, it can be considered that the formation reactions of the high MW components (Scheme 5), which definitely require molecular collisions, are suppressed by the cage effect of the complex cation, making the competing vanillin production relatively more favorable.

As shown in Scheme 5, there exists a relatively minor pathway in the degradation route of **VE**_**β**_, through which vanillyl alcohol and vanillic acid are formed via the disproportionation reaction of the vanillin residue followed by the ether cleavage. Comparing entries 2 and 4 in Table 1, it is evident that the yields of vanillyl alcohol and vanillic acid were hardly affected by the addition of **15C5** (with **15C5** addition, they were 9 and 2 mol % respectively, compared to 9 and 1 mol % without addition). From these results, it appears that, unlike the previous case with veratraldehyde,^24^ **15C5** does not seem to influence the disproportionation reaction of the vanillin residue in **VE**_**β**_. However, considering that the addition of **15C5** suppresses the polymerization pathway, another perspective is possible. As already discussed, the elimination reaction of vanillin from high MW components is less likely to proceed, and vanillin is thought to be produced from a sort of compounds where the A-ring side-chain and the B-ring are connected by an ether bond, such as **VE**_**β**_ and **VE**_**γ**_. Therefore, the yields of vanillyl alcohol and vanillic acid, which are produced after the disproportionation of the aldehyde group in the B-ring, are expected to increase in the **15C5**-added system where the formation of high MW components is less favored. The validity of this idea is also supported by the fact that the yields of vanillyl alcohol and vanillic acid tend to decrease in the systems with the addition of **TEG**, which instead promotes the formation of high MW components. Hence, the fact that the yields of vanillyl alcohol and vanillic acid remain at the same level as the no-additive case in the **15C5**-added system suggests that the reaction process required for the formation of these compounds, namely the disproportionation reaction of the aldehyde group, is suppressed in the **15C5**-added system.

#### Effects of **12C4** and **18C6**—Differences between the Model Compound **VE**_**β**_ and Lignin

In our previous study, we reported that the addition of crown ethers other than **15C5**, such as **12C4** and **18C6** (Scheme 1), resulted in an increase in the yield of vanillin during the alkaline oxidative degradation of native lignin in softwood flour.^24^ Table 2 summarizes the effects of various additives on the yield of vanillin from softwood flour in that study. On the other hand, no such increase in vanillin yield was observed with the addition of **TEG** or 1,4-dioxane. From these findings, it has been revealed that only ether compounds with a high inclusion ability for Na^+^ exhibit an effect of increasing vanillin yield, leading us to propose a mechanism of action for complex cations, including the cage effect discussed earlier. As summarized in Table 2, the effects of **15C5** addition on the degradation of **VE**_**β**_ appear to be generally consistent with the results from the previous study on softwood flour. In this section, we report the results of examining the effects of adding crown ethers with different ring sizes, **12C4** and **18C6**, on the degradation of **VE**_**β**_. Note that the degradation of **VE**_**β**_ was conducted under a nitrogen atmosphere, whereas the previous experiments using wood flour were carried out under aerobic conditions.^24^ Due to this difference in reaction atmosphere, the comparison in Table 2 is not intended to serve as a direct quantitative evaluation, but rather as a qualitative illustration of the differing reactivity trends upon the addition of the same crown ethers.

We conducted the alkaline degradation of **VE**_**β**_ for 4 h under nitrogen with the addition of either **12C4** or **18C6**. The results of these product yields are shown in entries 3 and 5 in Table 1. Unlike the results obtained with **15C5**, the yield of vanillin was found to decrease with the addition of these crown ethers compared to the pure NaOH system (vanillin yield: 46 mol %), with yields of 32 mol % (**12C4**-added system) and 34 mol % (**18C6**-added system). Additionally, the yield of vanillyl alcohol, which probably derive from the disproportionation products, also significantly decreased to 4 mol % in the **18C6**-added system and 3 mol % in the **12C4**-added system. The production of vanillic acid, another disproportionation-derived product, decreased to undetectable levels.

To further investigate these unexpected results, we conducted GPC analysis of the degradation products obtained. For the high MW components detected between elution times of 11–14 min, a significant increase was observed in the systems with the addition of **12C4** and **18C6**, especially in the **12C4**-added system (see Figure S5 in the Supporting Information for actual GPC chromatograms obtained for the **18C6**- and **12C4**-added systems). This led to a considerable increase in the total peak area corresponding to high MW components in the GPC chromatograms of these systems. These results suggest that in the systems with the addition of **12C4** or **18C6**, the formation of high MW components, which competes with the elimination of vanillin, was not suppressed as in the case of **15C5** addition, resulting in a decrease in vanillin yield. The decrease in the yields of vanillyl alcohol and vanillic acid observed in the systems with the addition of **12C4** and **18C6** also corresponds to the increase in high MW components.

The above results indicate that, although **12C4** and **18C6** increase the yield of vanillin from wood flour, as shown in Table 2, they do not positively affect the elimination of vanillin from the model compound **VE**_**β**_. The reason for the difference in reaction behaviors between the model system and the real system remains unclear. Actual lignin contains phenolic structures that produce vanillin through entirely different reaction pathways than Scheme 2,^29,41–46^ as well as various interunit linkages other than β-*O*-4. It is therefore natural to consider that the production of vanillin from actual lignin is far more complex than from **VE**_**β**_ and vanillin generated through the alkaline oxidative degradation of wood flour has various sources. Further investigation into the different effects of complex cations on **VE**_**β**_ and wood flour requires broader consideration, keeping in mind these aspects.

Even when limiting the discussion to the effect of complex cations on the degradation of **VE**_**β**_, it is necessary to pay attention to how the ease of interaction between Na^+^ and crown ethers is greatly influenced by the size of the crown ether ring.^36,37^ In the experiments of this paper, following our previous study,^24^ the amount of crown ether added was unified to a quarter molar equivalent of OH^–^. Thus, the concentration of complex cations present in the reaction system of this study differs depending on the type of crown ether added (**12C4**, **15C5**, **18C6**). Moreover, the chemical structure of the resulting complex cations varies with the crown ether. In particular, **12C4** is known to form a 2:1 sandwich-type complex cation with Na^+^ due to its smaller ring size.^47,48,49^ From this, it can be stated that complex cations with different chemical structures are generated at different concentrations in the reaction solution depending on the type of crown ether added. Hence, whether the complex cations derived from **12C4** and **18C6**, which had little effect in this paper, would exhibit similar behavior under a broader range of reaction conditions cannot be judged from the studies conducted in this study alone. To comprehensively understand the effect of complex cations on the vanillin elimination reaction, future systematic experiments are needed, varying factors such as metal ion concentration, type of metal ion, and amount of crown ether added.

## Conclusions

In this study, we have examined the details of the vanillin elimination reaction from vanillin end groups. For this examination, we employed **VE**_**β**_ (Scheme 4), which is closer to the actual lignin structure than veratraldehyde previously employed as a model for vanillin end groups. It has been found that **VE**_**β**_ forms an equilibrium mixture composed of compounds where the vanillin residue has migrated to other carbons on the side-chain, simply by dissolving in an alkaline solution. When this mixture is heated, the ether bond between the A-ring side-chain and the B-ring cleaves, resulting in the production of vanillin. Although the details of the mechanism remain unclear, it was further revealed that the reaction producing high MW components compete with this vanillin elimination.

In the degradation of **VE**_**β**_, the presence of complex cations formed between **15C5** and Na^+^ in the reaction solution suppressed the formation of high MW components and increased the yield of vanillin. One plausible reason for this, based on insights from our previous research, is that the cage effect caused by complex cations suppressed the formation reactions of high MW components, which require molecular collisions, making the competing vanillin production relatively more favorable. On the other hand, the addition of **12C4** and **18C6**, which improved the yield of vanillin in the alkaline oxidative degradation of wood flour, was found not to increase the vanillin yield from **VE**_**β**_. The cause of the difference in the effects of crown ether addition between the model system and the real system is currently unclear, but it may be related to the production of vanillin from phenolic structures and substructures other than β-*O*-4, which may only occur in the case of actual lignin molecules. These findings suggest that mechanistic insights obtained from model compounds are not necessarily directly applicable to native lignin systems, highlighting the need to consider the structural and chemical complexity of the actual reaction environment. Furthermore, the understanding of such differences, as revealed in this study, provides important fundamental knowledge for the development of future strategies to optimize lignin conversion processes.

The process of vanillin molecule elimination from the vanillin end groups, being the final stage in vanillin production, is relatively simple compared to the complex oxidation reactions involved in the preceding stages (Scheme 2). Additionally, the selectivity of vanillin elimination in this reaction is directly linked to the overall yield of vanillin in the process. The yield of vanillin from **VE**_**β**_, without the addition of any additives, is at most 50 mol %. This suggests that the selectivity of vanillin elimination from vanillin end groups produced in actual lignin molecules (Scheme 2) is not necessarily high. In this regard, the demonstration in this study that the vanillin elimination process can be controlled by the addition of **15C5** (presence of **15C5**–Na^+^ complex cations) is significant. From the perspectives of ease of examining reaction control methods based on reaction mechanisms and the magnitude of the effect when control is successful, the vanillin elimination pathway will likely be a route examined in more detail from a molecular standpoint in the future.

## Supplementary Material



## Notes

Isolation and analysis of **VE**_**γ**_ and **ArG** (Figures S1–S3); two proposed mechanisms for vanillin elimination (Scheme S1); disproportionation pathway in the alkaline degradation of **VE**_**β**_ (Figure S4); GPC chromatograms of acetylated mixtures with **18C6** or **12C4** (Figure S5); extended version of Table 1 (Table S1); synthetic routes of model compounds (Schemes S2–S4) (PDF)
