# Supplementary material for: Alkaline-Induced Degradation Pathways of β‑O‑4-Linked Vanillin Moieties Produced during Lignin Oxidation and the Effect of Na+‑Cyclic Polyether Complexes
Source: ACS Omega. 2025 Aug 22;10(35):40646–57. doi: 10.1021/acsomega.5c07658 (PMC12423867; doi:10.1021/acsomega.5c07658)
Supplement: Supplementary file 1 [file ao5c07658_si_001.pdf]

Supporting Information

Alkaline-Induced Degradation Pathways of β-O-4-Linked Vanillin Moieties Produced During Lignin Oxidation and the Effect of Na^+-Cyclic Polyether Complexes

Yuki Hirano, Takashi Hosoya*, Hisashi Miyafuji

Graduate School of Life and Environmental Sciencesm Kyoto Prefectural University. 1-5 Shimogamo-hangi-cho, Sakyo-ku, Kyoto, 606-8522, Japan

* Corresponding author: hosoya_t@kpu.ac.jp

Contents

Isolation and analysis of **VE**$_\gamma$ and **ArG** (Figure S1−S3)
Pages S2−S7

Two proposed mechanisms for the elimination of vanillin (Scheme S1)
Page S8

Disproportionation pathway in the alkaline degradation of **VE**$_\beta$ (Figure S4)
Page S9−S10

GPC Chromatograms of the acetylated reaction mixtures derived from the degradation of **VE**$_\beta$ in the presence of **18C6** or **12C4** (Figure S5)
Page S11

Extended version of Table 1 (Table S1)
Page S12

Synthetic routes of model compounds (Scheme S2−S4)
Pages S13−S20

Number of pages: 20
Number of figures: 5
Number of schemes: 4
Number of tables: 1

Isolation and analysis of VE_γ and ArG

When aiming for product isolation, 30 mg of the starting material, **VE_β**, was utilized, and the degradation experiment of **VE_β** was conducted using the same method as described in the experimental section of the text. After the reaction, hydrochloric acid (10 %) was added to the test tube at room temperature to adjust the pH of the solution to approximately 1. The organic layer obtained by extracting this solution with ethyl acetate was washed once with brine and then dried under reduced pressure. Subsequently, it was dissolved in a solution of 0.1% trifluoroacetic acid in water/acetonitrile (9/1, approximately 1 mL) and used as a sample for the following automated flash chromatography. The chromatography was performed using the single channel automated flash chromatography (Smart Flash EPCLC AI-580S) from Yamazen Corporation. The separation conditions were as follows: column (ODS-SM, Uni. Premium L, 40 g), peak collection mode, detection wavelength $UV_{254\ nm}$, flow rate 20 mL/min, and a solution of 0.1% trifluoroacetic acid in water/acetonitrile.

The compound recovered as the eluent dilution solution was dried under reduced pressure, dissolved in chloroform-d ($CDCl_3$), and subjected to ^1H NMR analysis. After removing $CDCl_3$ under reduced pressure, it was acetylated with anhydrous acetic acid (0.2 mL)/pyridine (0.2 mL), dissolved again in $CDCl_3$, and subjected to ^1H NMR analysis of the acetylated product. ^1H NMR spectra were measured at room temperature using a JNM-ECZ 400S (400 MHz) spectrometer, and chemical shifts (ppm) were measured relative to chloroform (δ_H = 7.26 ppm). The following abbreviations were used in the ^1H NMR spectrum: s = singlet, d = doublet, t = triplet, q = quartet, m = multiplet, dd = doublet of doublets.

Identification of VE_γ

The compound detected at a retention time of 22.3 min in the HPLC analysis of the sample after degradation under the conditions of 0 h reaction time for **VE_β** (Figure 2A) was isolated and subjected to ^1H NMR analysis. The spectrum obtained as a result is shown in Figure S1A, and it was analyzed by comparing it with the ^1H NMR spectrum of **VE_β** shown in Figure S2A.

In the spectrum of the isolated compound shown in Figure S1A, a singlet corresponding to one proton at 9.85 ppm was detected, indicating the presence of an aldehyde group in this compound. Focusing on the chemical shift region where the aromatic protons appear, signals corresponding to six protons were detected at 6.83-7.43 ppm. Additionally, two singlets corresponding to three protons each, presumably associated with methoxy groups, were detected at 3.95 ppm and 3.86 ppm. Observing the protons of the C_3 side-chain appearing in the

region of 3.8-5.0 ppm, a doublet considered to be the α proton at 4.93 ppm, a multiplet considered to be the β proton at 4.21 ppm, and two γ protons were detected at 4.17-4.07 ppm. Furthermore, protons attributed to ethoxy groups appeared at 3.73 and 1.42 ppm, as quartets and triplets of two and three protons, respectively.

The above results collectively indicate that the isolated compound has a structure similar to **$VE_β$**, possessing two methoxy groups, one ethoxy group, an aldehyde group, and four protons on the C_3 side-chain. However, the chemical shifts of the detected proton signals differ slightly from those of **$VE_β$** in Figure S2A (δ = 9.85 ppm (s, 1H, CHO), 7.43-7.38, 7.02-6.82 ppm (m, 6H, Ar-H), 4.99 ppm (dd, J = 5.05 and 3.26 Hz, 1H, $C_α$-H), 4.39 ppm (m, 1H, $C_β$-H), 4.11 ppm (q, J = 7.11 Hz, 2H, OEt), 3.98-3.78 ppm (m, 2H, $C_γ$-H), 3.92 ppm (s, 3H, OMe), 3.80 ppm (s, 3H, OMe), 3.09 ppm (d, J = 3.50 Hz, OH), 2.55 ppm (t, J = 6.47 Hz, OH), 1.45 ppm (t, J = 6.82 ppm, 3H, OEt)). Therefore, we hypothesized that the isolated compound is a derivative of **$VE_β$**, with the B-ring of **$VE_β$** having rearranged to either the α- or γ- position of the C_3 side-chain.

Based on this hypothesis, we performed acetylation of hydroxyl groups in the isolated compound to identify the position of the B-ring rearrangement in this compound. Upon comparison of the spectrum of the acetylated compound with that before acetylation, it was expected that the signals of protons bonded to carbons bearing hydroxyl groups would shift to the lower magnetic field due to the introduction of acetyl groups. The ^{1}H NMR spectrum of the acetylated compound and the assignment of each signal were as shown in Figure S1B. In this spectrum, a singlet presumably representing the aldehyde proton was detected at 9.83 ppm, and signals corresponding to six protons derived from aromatic rings were detected at 7.42-6.82 ppm. Additionally, two singlets corresponding to three protons each, presumed to be associated with methoxy groups, were detected at 3.88 ppm and 3.85 ppm. Furthermore, doublets considered to be α protons at 6.08 ppm, multiplets considered to be β protons at 5.60 ppm, and doublets of doublets corresponding to two γ protons were detected at 4.18-6.08 ppm. Protons derived from ethoxy groups were detected at 4.04 ppm and 1.43 ppm, and singlets corresponding to acetyl groups were detected at 2.10 ppm and 2.01 ppm. Peaks attributable to impurities introduced maybe during acetylation were detected at 5.13-5.29 ppm and 2.55 ppm, but these signals were ignored. Consequently, the signals of α and β protons in the acetylated compound (6.08 ppm and 5.60 ppm, respectively) were shifted to the lower magnetic field by 1.14 ppm and 1.40 ppm, respectively, compared to those of the compound before acetylation (4.94 ppm and 4.20 ppm). However, the shifts for the two γ protons due to acetylation were only 0.1 ppm and 0.09 ppm, respectively. This suggests that the γ position of the isolated compound does not bear a hydroxyl group but is instead bound to a vanillin residue, indicating

that this compound is **VE**$_{\gamma}$.

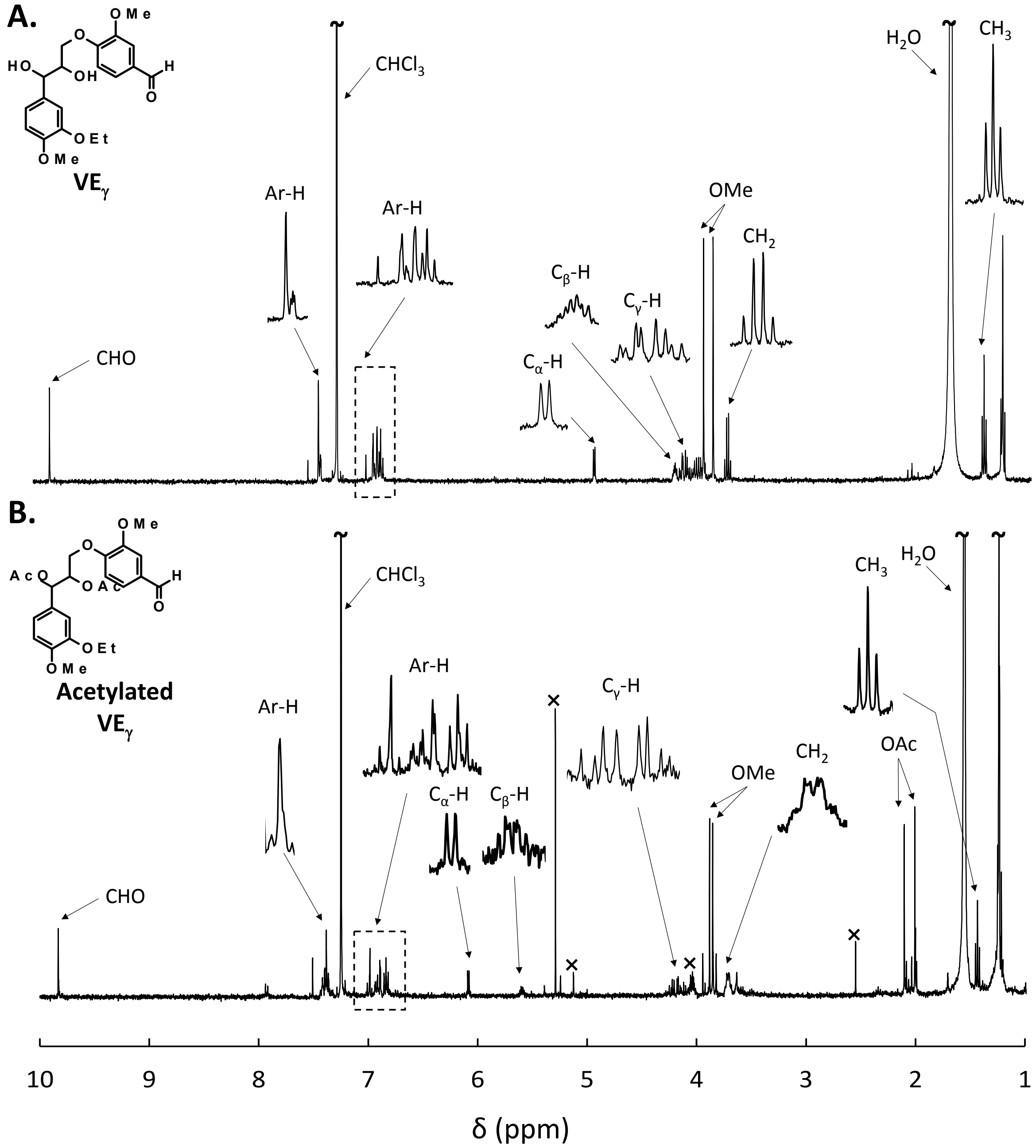

Figure S1. ^{1}H NMR spectra of **VE**$_{\gamma}$ (A), acetylated **VE**$_{\gamma}$ (B) in $CDCl_3$ (400 MHz). The signals indicated with "×" in B are derived from impurities probably from the acetylation process.

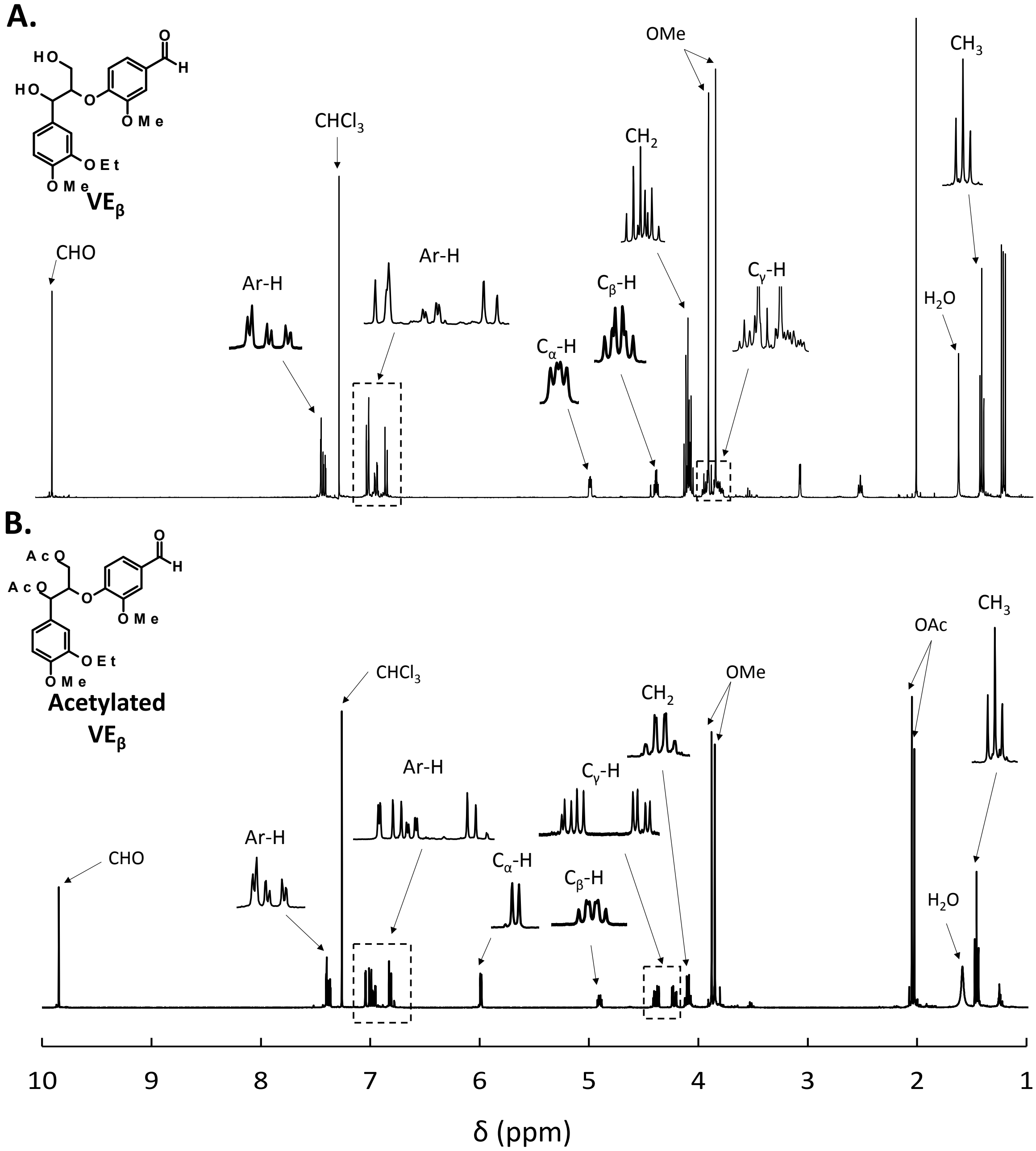

Figure S2. ^{1}H NMR spectrum of $\mathbf{VE_\beta}$ (A) and acetylated $\mathbf{VE_\beta}$ (B) in $CDCl_3$ (400 MHz).

To further confirm the validity of the above discussion, we compared the ^{1}H NMR spectra of synthesized $\mathbf{VE_\beta}$ before and after acetylation, as shown in Figures S2A and S2B. The chemical shift of the α proton was significantly shifted to the lower magnetic field due to acetylation (before acetylation: 4.99 ppm, after acetylation: 5.99 ppm), corresponding to the presence of a hydroxyl group at the α-position. The chemical shift of the two γ protons (3.98-3.78 ppm) also shifted to the lower magnetic field considerably due to acetylation (4.39-4.22 ppm), although not as much as observed for the α proton. This phenomenon was not observed during the acetylation of the compound presumed to be $\mathbf{VE_\gamma}$, suggesting that $\mathbf{VE_\beta}$ possesses a

hydroxyl group at the γ position. Additionally, in the case of **$VE_β$**, a shift to a lower magnetic field due to acetylation was also observed in the chemical shift of the β proton (before acetylation: 4.39 ppm, after acetylation: 4.90 ppm). However, this shift amount (0.51 ppm) is significantly smaller than the shift amount observed in the β position in the case of **$VE_γ$** mentioned above (1.40 ppm). This small shift to a lower magnetic field of the β proton in the acetylation of **$VE_β$** is likely due to the β proton in **$VE_β$** being sandwiched between acetyl groups. Taken together, all these results support the conclusion that the compound isolated from the mixture at 0 h of reaction time is **$VE_γ$** with the vanillin residue having rearranged to the γ position.

Based on the assumption that the isolated compound is **$VE_γ$**, we compared the coupling constant of the α proton (J = 5.32 Hz) with that of **$VE_β$** (J = 5.02 Hz) and the literature value (J = 5.1 Hz) (Marcelle et al, 1989). As a result, the isolated compound was estimated to be the *erythro* form of **$VE_γ$**.

*Identification of **ArG***

After the degradation of **$VE_β$** for 4 h, a compound detected at a retention time of 8.1 min in the HPLC analysis of the sample was isolated (Figure 2B), and ^{1}H NMR analysis was conducted. The result of the ^{1}H NMR analysis yielded the spectrum shown in Figure S3A. Due to the limited amount of isolated compound, there were signals with unclear shapes and signals buried in the background, making them difficult to discern. However, signals at 6.95-6.81 ppm (3H), 4.79 ppm (d, J = 5.89 Hz, 1H), 4.09 ppm (2H), 3.87 ppm (s, 3H), 3.86-3.79 ppm and 3.74 ppm (3H), and 1.46 ppm (t, J = 7.09 Hz, 3H) were identified. For signals where the shape was not clearly discernible, or coupling constants were not determined, these details were not provided. When comparing this result with the ^{1}H NMR analysis of **VGL** synthesized in our previous study (Hirano et al. 2022), except for the presence of the ethoxy group peaks (4.09 ppm and 1.46 ppm in Figure S3A), the isolated compound and **VGL** showed almost identical chemical shift signals. Particularly, peaks corresponding to the α, β, and γ protons in the spectrum of **VGL**, which are important, at 4.67 ppm, 3.78 ppm, 3.64 ppm, and 3.52 ppm, respectively, were also detected in Figure S3A (the peak presumed to be the β position in Figure S3A is buried under the peak of one of the γ protons detected at 3.86-3.79 ppm). Therefore, based on the above findings, the isolated compound represented by the spectrum in Figure S3A is estimated to be a compound where the 3rd methoxy group of **VGL** is replaced with an ethoxy group, referred to as **ArG**.

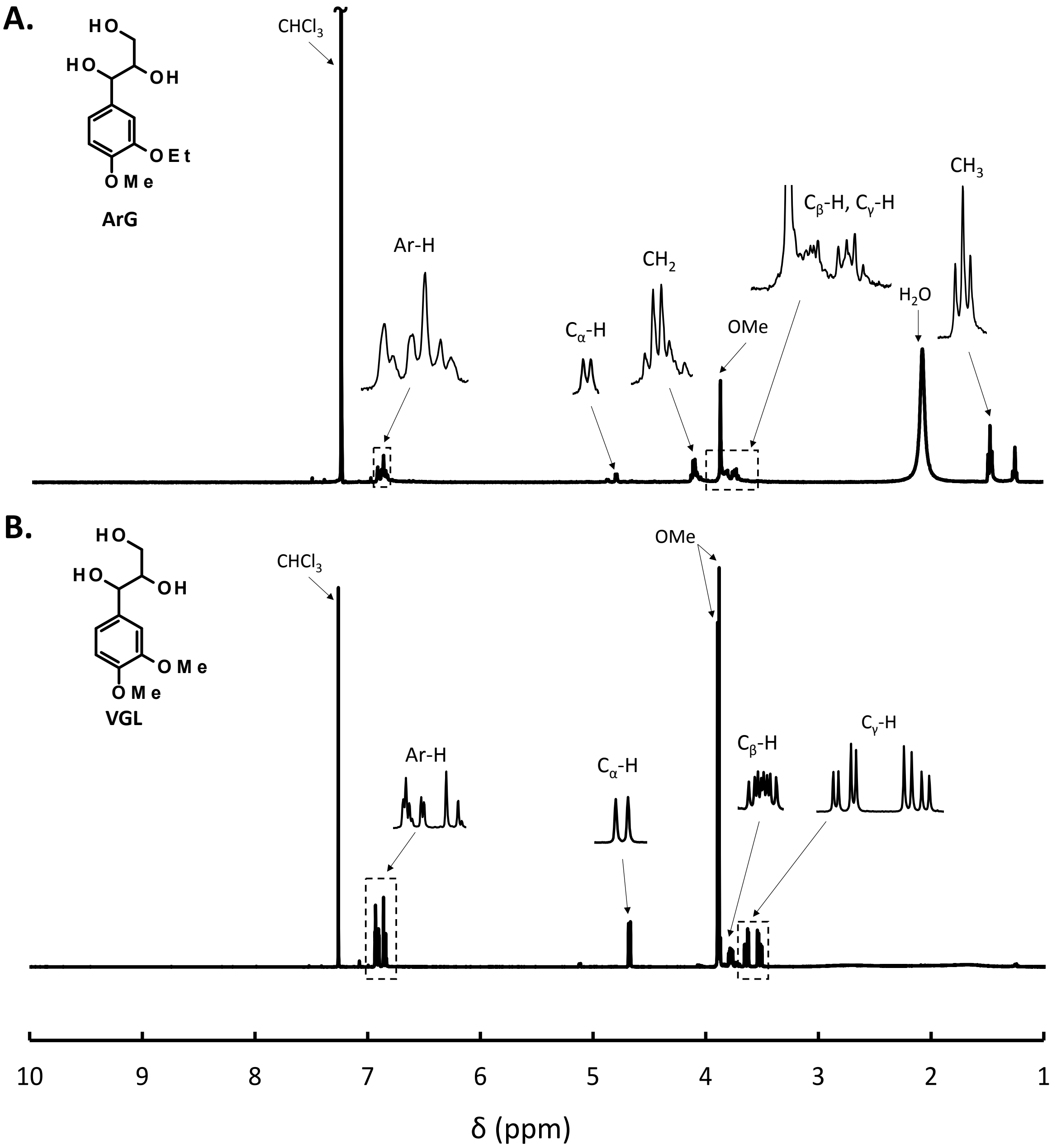

Figure S3. ^{1}H NMR spectra of **ArG** (A), and **VGL** (B) in $CDCl_3$ (400 MHz).

Two proposed mechanisms for the elimination of vanillin

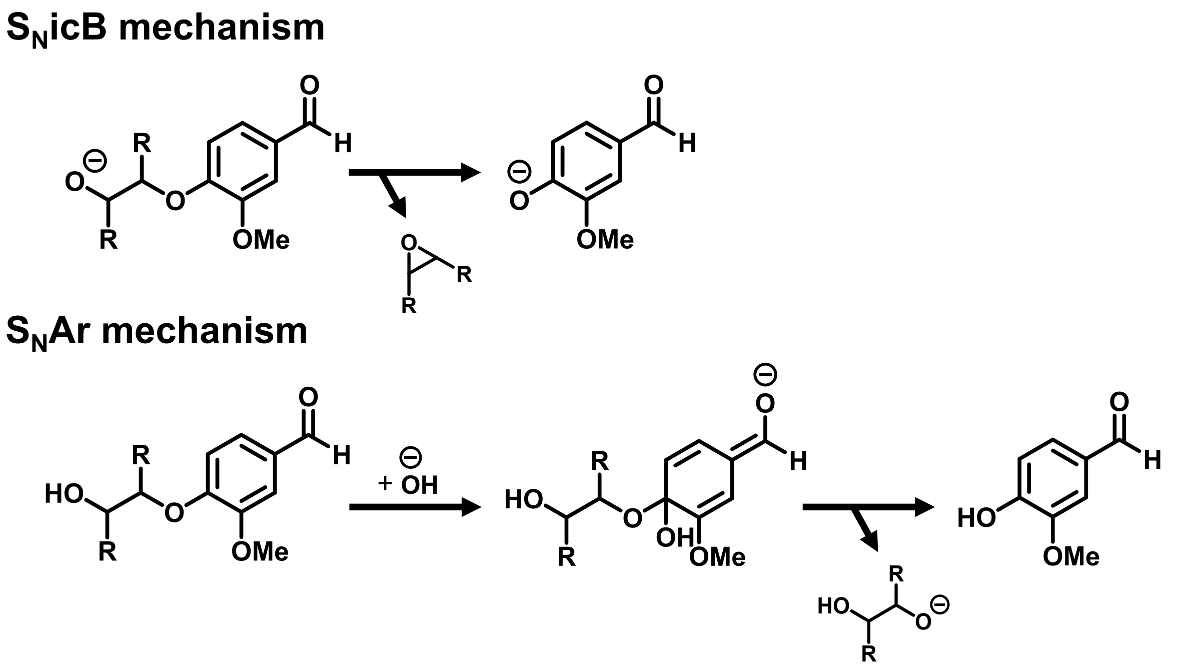

Scheme S1. The $S_N$icB and S_NAr mechanisms for vanillin elimination.

As noted in the main text, two mechanisms have been proposed for the elimination of the vanillin residue: the $S_N$icB mechanism and the S_NAr mechanism. Here, we provide a brief explanation of these mechanisms. As illustrated in Scheme S1 above, the $S_N$icB mechanism involves the nucleophilic attack by a neighboring oxyanion on the benzylic carbon bearing the ether linkage, leading to the elimination of the vanillin molecule. In contrast, the S_NAr mechanism is initiated by the nucleophilic attack of a hydroxide ion on the C_4 position of the vanillin residue. To date, no conclusive evidence has been presented to fully confirm the validity of either of these proposed pathways.

The rearrangement reactions and the vanillin elimination via the S_NAr mechanism share similarities in terms of the nature of the nucleophile and the site of attack. Thus, the observation that the rearrangement reactions readily proceed at room temperature seemingly implies that vanillin elimination occurs via the S_NAr mechanism. However, vanillin elimination via the S_NAr mechanism requires the attack of OH^- on the vanillin residue rather than the intramolecular oxyanion, yet no evidence supporting the occurrence of such intermolecular reactions was found in this investigation. The necessity of heating for vanillin production from **VE**$_\beta$ suggests that the mechanisms underlying vanillin elimination and the rearrangement of the vanillin residues may differ, potentially lending support to the $S_N$icB mechanism in this scenario. Nevertheless, given that the data from this study could align with either the S_NAr or $S_N$icB mechanisms, it underscores the need for more focused research to clarify the specific reaction mechanisms governing the ether cleavage.

Disproportionation pathway in the alkaline degradation of $VE_β$

To verify the presence of a disproportionation pathway in the degradation of **$VE_β$**, we synthesized **D_1** and **D_2** and investigated their retention times in the HPLC analysis. The results showed that neither of these compounds was detected among the degradation products at 0 and 4 h, as indicated by the HPLC chromatograms in Figures 2A and 2B. Additionally, no significant peaks were observed around the retention times of **D_1** and **D_2**, suggesting that disproportionation products derived from **$VE_α$** and **$VE_γ$** were not present in the reaction mixture at detectable concentrations either. On the other hand, vanillyl alcohol and vanillic acid, which are believed to be formed via the cleavage of the ether bonds in **D_1** and **D_2**, were detected in low yields of 9 and 1 mol%, respectively, after heating **$VE_β$** for 4 h (Entry 2 in Table 1). In experiments where the heating time was reduced from 4 h to 2 h, **D_1** and **D_2** were detected by HPLC, albeit in very small amounts (see Figure S4 in the Supporting Information). Additionally, at a reaction time of 2 h, numerous small peaks were observed around the retention times of these compounds, which may correspond to disproportionation products derived from the rearranged equilibrium components. Due to the limited scale of the reaction, the amounts of these minor products were too small to allow for isolation and structural characterization. The synthesis of standard samples of rearranged products bearing vanillyl alcohol or vanillic acid residues at the α- or γ-position is also not straightforward. Based on these considerations, further analysis and identification of these peaks were not conducted in this study.

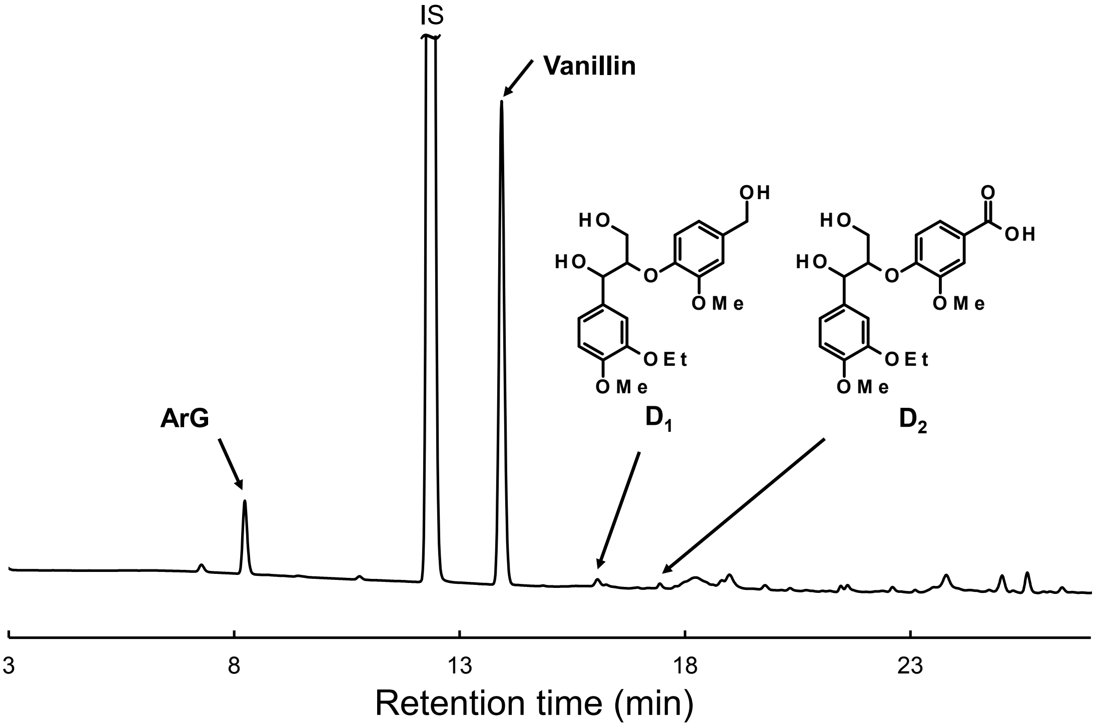

Figure S4. HPLC chromatograms of reaction mixtures obtained from the degradation of **$VE_β$** for 2 h in 4.0 mol/L NaOH aq. at 120 °C under N_2. Detection wavelength: UV_{280nm}.

These results suggest that, in the degradation of **VE$_\beta$** under the conditions of 4.0 mol/L NaOH aq./120°C/N_2, the disproportionation reaction of the vanillin residue indeed occurs and succeeding ether bond cleavage results in the formation of vanillyl alcohol and vanillic acid. However, such a disproportionation pathway in **VE$_\beta$** is not as pronounced as in the case of veratraldehyde, as indicated by the significantly higher yield of vanillin compared to vanillyl alcohol, as shown in Entry 2 in Table 1. This is supported by the considerable stability of vanillyl alcohol under the conditions of this paper (recovery rate after 4 h of reaction of the compound: 80 %). In other words, while the formation of vanillin from veratraldehyde is slow enough to compete with its disproportionation reaction, in the case of **VE$_\beta$** and its rearranged products, the vanillin formation reaction occurs considerably faster than the disproportionation reaction. It should be noted that there is a significant difference between the yields of vanillyl alcohol and vanillic acid, the final products of the disproportionation pathway, which might be attributed to the differences in the rates and selectivity of the ether bond cleavage following the disproportionation.

GPC Chromatograms of the acetylated reaction mixtures derived from the degradation of VE$_\beta$ in the presence of 18C6 or 12C4

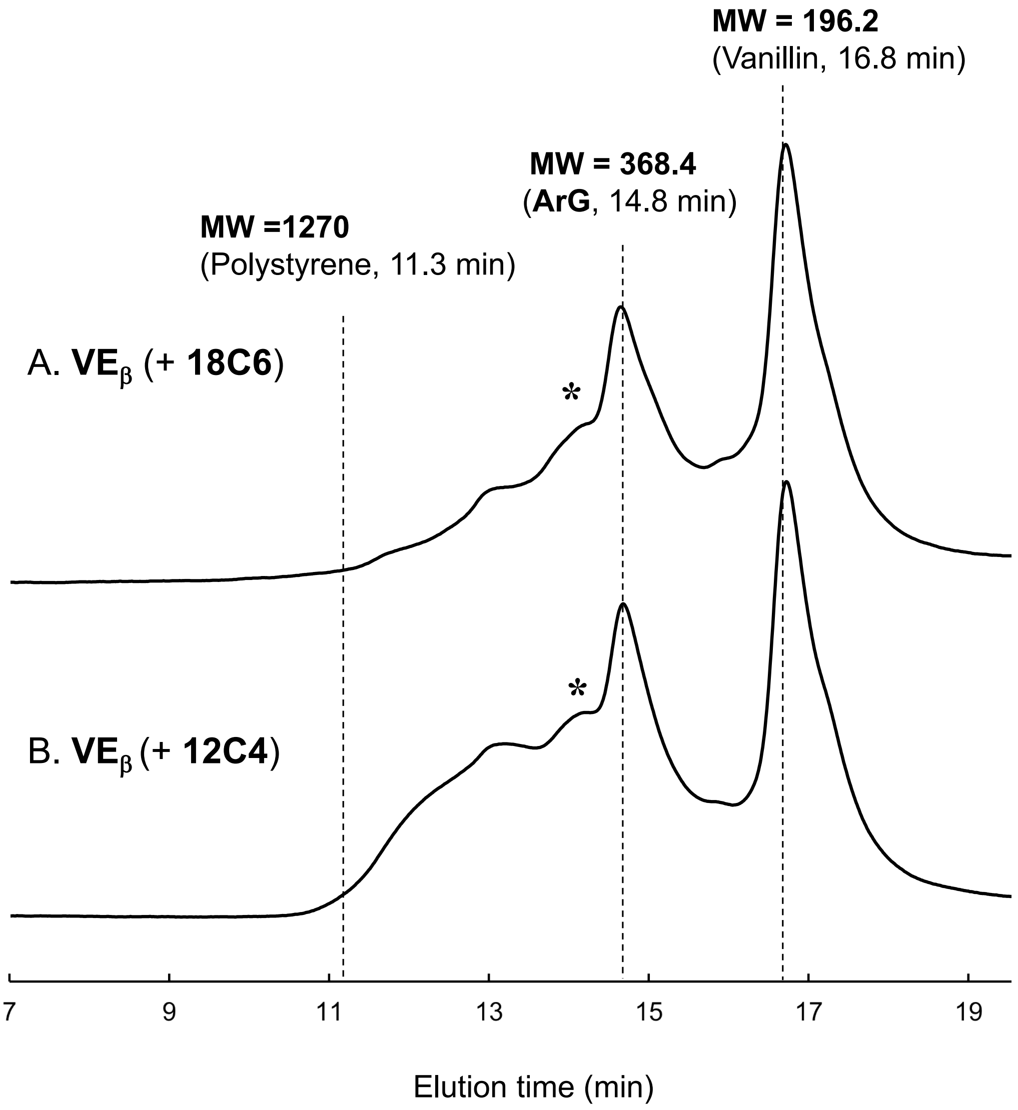

Figure S5. GPC Chromatograms of the acetylated reaction mixtures derived from the degradation of **VE$_\beta$** in the presence of 1**8C6** (A), **12C4** (B) in 4.0 mol/L NaOH aq. at 120°C for 4 h under N_2. Detection wavelength: $UV_{280\ nm}$. The molecular weights of several acetylated authentic compounds were provided along with their elution times. Peaks at 14.5 min, which are mentioned in the text, are indicated with an asterisk.

The peak at 14.5 min tends to decrease in the presence of complex cations, as was also evident in the **15C5**-added system. In the main text, this peak is discussed as part of the high molecular weight fraction; however, it is possible that some of the compounds contributing to this peak are selectively suppressed in the presence of inclusion cations.

Extended version of Table 1

Table S1. Product yields and recovery of the starting material after the alkaline degradation of $\mathbf{VE}_\beta$ and **VG** in 4.0 mol/L NaOH aq. at 120 ºC under N_2 (Extended version of Table 1).

Entry	Starting material	Medium	Reaction time (h)	Recovery (%)	Produc yield (mol%)[a]						
					$\mathbf{VE}_\gamma$	**ArG**	Vanillin	Vanillyl alcohol	Vanillic acid	**VGL**	Guaiacol
1	$\mathbf{VE}_\beta$	NaOH aq.	0	76	17	ND[b]	1	ND	ND	—	—
2	$\mathbf{VE}_\beta$	NaOH aq.	4	0.5[c] [0.5,0.4] [c]	ND	47 [47,47]	46 [46,45]	9 [8,10]	1 [1,1]	—	—
3	$\mathbf{VE}_\beta$	NaOH aq.+**18C6**	4	ND	ND	48 [47,49]	34 [34,33]	4 [4,3]	ND	—	—
4	$\mathbf{VE}_\beta$	NaOH aq.+**15C5**	4	ND	ND	67 [67,67]	63 [63,63]	9 [8,9]	2 [1,2]	—	—
5	$\mathbf{VE}_\beta$	NaOH aq.+**12C4**	4	ND	ND	43 [42,43]	32 [32,32]	3 [3,2]	ND	—	—
6	$\mathbf{VE}_\beta$	NaOH aq.+**TEG**	4	1	ND	42	33	4	ND	—	—
7	$\mathbf{VE}_\beta$	NaOH aq. + 1,4-dioxiane	4	0.9 [0.6,1.2]	ND	49 [50,47]	47 [46,47]	9 [11,6]	1 [1,0.5]	—	—
8	**VG**	NaOH aq.	4	27		—	—	—	—	44 (60)[d]	64 (88)

[a] Product yield is based on the initial molar amount of the starting material. [b] ND: not detected. [c] For the items accompanied by brackets, the same experiment was conducted twice. The values inside the brackets indicate the yields obtained in each experiment, while the value outside the brackets represents the average yield from the two experiments. [d] The number in the parenthesis shows yields (mol%) of **VGL** or guaiacol on the basis of the degraded **VG**.

Synthesis of model compounds

General information

All synthetic reactions were monitored using TLC on silica gel plates, with a solvent system consisting of *n*-hexane and ethyl acetate as the eluent. Spot visualization was achieved using a 10 wt% solution of phosphomolybdic acid in ethanol and UV detection at 254 nm unless otherwise specified. Column chromatography was performed using CHROMATOREX PSQ100B silica gel (Fuji Silysia Chemical Ltd.), with the amount of silica gel adjusted to approximately 50 g per 1 g of crude mixture. NMR spectra were recorded on a JNM-ECZ 400S spectrometer (400 MHz for ^1H and 100 MHz for ^{13}C) in $CDCl_3$ at room temperature, with chemical shifts (ppm) referenced to chloroform (δ_H = 7.26 ppm, δ_C = 77.0 ppm). The following abbreviations were used in the ^1H NMR spectra: s = singlet, d = doublet, t = triplet, q = quartet, m = multiplet, dd = doublet of doublets, dt = doublet of triplets.

Synthesis of **VE$_\beta$**

VE$_\beta$, a model compound for the aldehyde end of coniferous lignin, was synthesized in five steps starting from vanillin, following the pathway illustrated in Scheme S2.

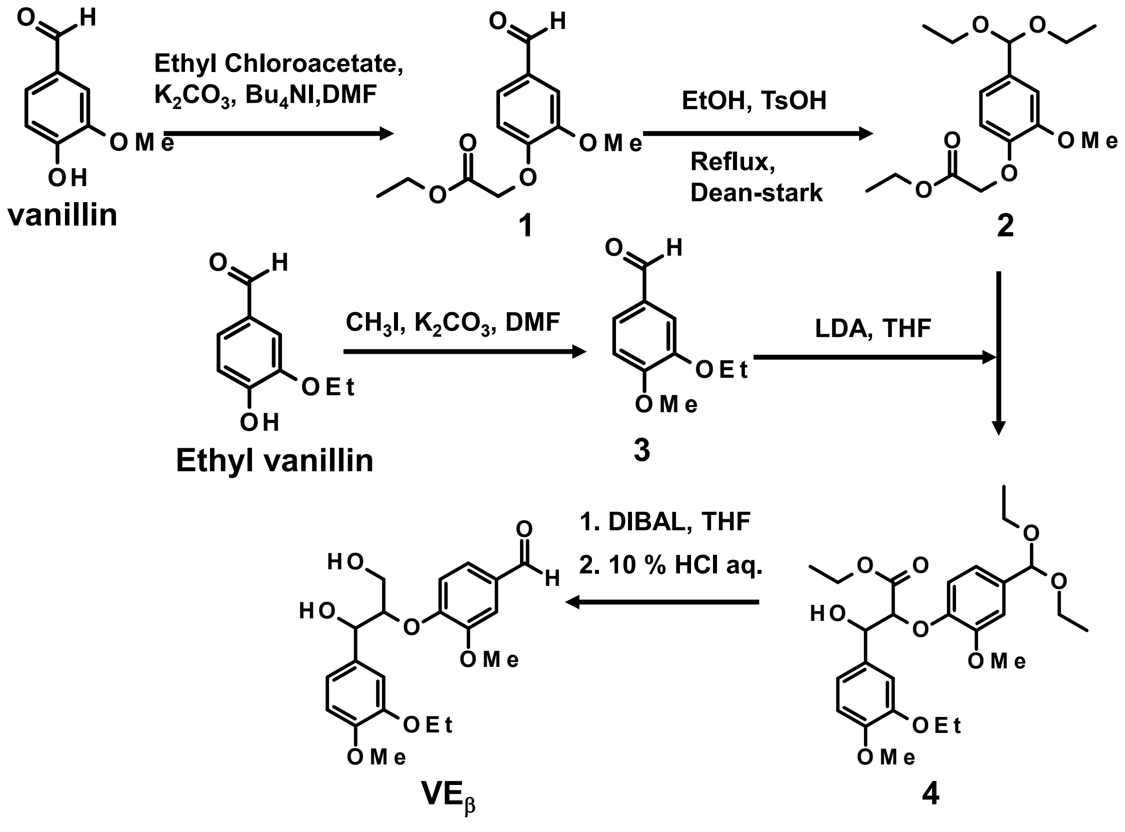

Scheme S2. Synthetic route for **VE$_\beta$**

Synthesis of compound **1**

Vanillin (10.0 g, 65.7 mmol), potassium carbonate (18.2 g, 131.7 mmol), and tetra-*n*-butylammonium iodide (2.4 g, 6.5 mmol) were dissolved in DMF (60 mL), and ethyl chloroacetate (8.4 mL, 78.8 mmol) was added. The mixture was stirred at 60°C. After 2 h, the reaction mixture was extracted with ethyl acetate/hexane (1:5) mixed solvent, and the organic layer was washed three times with brine and dried over anhydrous sodium sulfate. After removal of the solvent under reduced pressure, compound **1** (14.9 g, 95%) was obtained as yellow crystals. ^{1}H NMR (400 MHz, $CDCl_3$) δ = 9.86 (s, 1H), 7.45-7.40 (m, 2H), 6.87 (d, J = 8.04 Hz, 1H), 4.78 (s, 2H), 4.27 (q, J = 7.06 Hz, 2H), 3.95 (s, 3H), 1.29 (t, J = 6.94 Hz, 3H) ppm. ^{13}C NMR (100 MHz, $CDCl_3$) δ = 191, 168, 152, 150, 131, 126, 112, 109, 66, 61, 56, 14 ppm.

Synthesis of compound **2**

Compound **1** (0.85 g, 3.6 mmol) was dissolved in ethanol (15.0 mL), and *p*-toluenesulfonic acid monohydrate (0.06 g, 0.36 mmol) was added. The mixture was refluxed at 110°C. During the reaction, water generated was removed using a Dean-Stark apparatus. After 2 h, the reaction mixture was cooled on ice, and sodium bicarbonate powder was slowly added to adjust the pH of the reaction mixture to 7. The reaction mixture was then reduced to approximately half its original volume under reduced pressure. It was then extracted with ethyl acetate, and the organic layer was washed once with saturated sodium bicarbonate solution and once with brine. After drying over anhydrous sodium sulfate, the solvent was removed under reduced pressure. The residue was purified by column chromatography (ethyl acetate/hexane = 1/2), yielding compound **2** (0.78 g, 69%) as a colorless oily substance.^{1}H NMR (400 MHz, $CDCl_3$) δ = 7.04 (s, 1H), 6.96(d, *J* = 8.06 Hz, 1H), 6.78 (d, *J* = 8.17 Hz, 1H), 5.42 (s, 1H), 4.67(s, 2H), 4.25 (q, *J* = 7.42 Hz, 2H), 3.89(s, 3H), 3.65~3.56(m, 2H), 3.56~3.46(m, 2H), 1.3~1.2 (m, 9H) ppm. ^{13}C NMR (100 MHz, $CDCl_3$) δ = 169.0, 149.5, 147.2, 133.5, 119.0, 113.6, 110.2, 101.5, 66.6, 61.2, 55.9, 15.2, 14.2 ppm.

Synthesis of compound **3**

Ethyl vanillin (2.0 g, 12.0 mmol) and potassium carbonate (2.5 g, 18.1 mmol) were dissolved in DMF, and methyl iodide (1.1 mL, 18.1 mmol) was added. The mixture was stirred at room temperature. The progress of the reaction was monitored using a 5% aqueous solution of iron(III) chloride. After 2 h, the reaction mixture was filtered, and the filtrate was extracted with ethyl acetate/hexane (5/1) solution. The organic layer was washed three times with brine and dried over anhydrous sodium sulfate. After removal of the solvent under reduced pressure, compound **3** (2.19 g, 101%) was obtained as white crystals. ^{1}H NMR (400 MHz, $CDCl_3$) δ = 9.84 (s, 1H),7.44 (dd, *J* = 1.95 and 8.19 Hz, 1H), 7.40(d, *J* = 1.79 Hz, 1H), 6.97(d, *J* = 8.11 Hz, 1H), 4.16 (q, *J* = 7.33 Hz, 2H) 3.96 (s, 3H), 1.49 (t, *J* = 7.19 Hz, 3H) ppm. ^{13}C NMR (100 MHz, $CDCl_3$) δ = 191.1, 154.7, 148.9, 130.1, 126.8, 110.5, 110.0, 64.4, 56.2, 14.7.ppm.

Synthesis of compound **4**

The glassware used in the experiment was dried overnight in a desiccator at 105 °C. Diisopropylamine and THF were each dried overnight over molecular sieves 4A. A reaction flask containing approximately 700 mg of powdered molecular sieves 3A and two separate flasks containing compound **2** and compound **3** were dried under vacuum on phosphorus pentoxide for 1.5 h. Diisopropylamine (0.71 mL) and THF were added to the reaction flask at room temperature. The reaction temperature was then lowered to -78 °C, and *n*-butyllithium (3.14 mL) was added dropwise over 5 min using a syringe. The temperature of the reaction mixture was controlled to not exceed 0 °C while gradually warming up. After stirring for 30 min, the temperature of the reaction mixture was again lowered to -78 °C, and compound **2** (0.79 g, 2.5 mmol), dissolved in THF, was added dropwise over 5 min using a syringe. Compound **3** (0.45 g, 2.5 mmol), also dissolved in THF, was then added dropwise over 15 min. After 1 h, the reaction mixture was brought back to room temperature, and saturated ammonium chloride solution was added to quench the reaction. The reaction mixture was extracted with ethyl acetate, and the organic layer was washed once with saturated sodium bicarbonate solution and once with brine. After drying over anhydrous sodium sulfate, the solvent was removed under reduced pressure. The residue was purified by column chromatography (ethyl acetate/hexane = 1/3→1/1), yielding compound **4** (0.48 g, 38.7%) as a pale yellow oil. ^{1}H NMR (400 MHz, $CDCl_3$) δ = 7.07~6.70 (m, 6H), 5.40 (s, 1H), 5.12 (t, *J* = 5.36 Hz, 1H), 4.70 (d, *J* = 6.05 Hz, 1H), 4.16~4.07 (m, 6H), 3.87 (s, 3H), 3.85 (s, 3H), 3.66~3.55 (m, 3H), 3.55~3.45 (m, 2H), 2.04 (s, 3H), 1.45 (t, *J* = 7.63 Hz, 3H), 1.28~1.19 (m, 9H), 1.14 (t, *J* = 7.15 Hz, 3H) ppm. ^{13}C NMR (100 MHz, $CDCl_3$) δ = 169.3, 150.52, 149.0, 148.0, 147.2, 135.0, 131.6, 119.4, 119.3,

118.2, 111.4, 110.9, 110.3, 101.4, 100.0, 91.7, 83.9, 73.8, 64.2, 61.3, 60.5, 56.0, 55.9, 21.1, 15.2, 14.8, 14.2, 14.1 ppm.

Synthesis of $\mathbf{VE}_\beta$

The glassware used in the experiment was dried overnight in a drying oven at 105 °C, and toluene was dried overnight over molecular sieves 4A. A reaction flask containing approximately 350 mg of powdered molecular sieves 3A and a separate flask containing compound **4** were dried under vacuum on phosphorus pentoxide for 1.5 h. Compound **4** (0.48 g, 0.98 mmol), dissolved in toluene, was added to the reaction flask at room temperature. Next, the reaction temperature was lowered to 0°C, and a solution of diisobutylaluminum hydride in toluene (2.0 mL, 3.0 mmol) was added dropwise over 15 min. After stirring for 1.5 h, ethanol and distilled water were added sequentially to quench the reaction. Hydrochloric acid (10%) was added to acidify the reaction mixture, followed by extraction with ethyl acetate. The organic layer was washed once with brine and dried over anhydrous sodium sulfate. The solvent was removed under reduced pressure, and the residue was purified by column chromatography (ethyl acetate/hexane = 1/1→2/1), yielding $\mathbf{VE}_\beta$ (0.15 g, 41.5%) as a colorless oil. $\mathbf{VE}_\beta$ was acetylated with anhydrous acetic acid/pyridine and analyzed by NMR. By comparing the results of the NMR analysis of the acetylated product with literature values, the ratio of the *erythro* and *threo* forms of the synthesized $\mathbf{VE}_\beta$ was estimated to be 1:0.09（Marcelle *et al*, 1989）. ^{1}H NMR (400 MHz, $CDCl_3$) δ = 9.84 (s, 1H), 7.41~7.35 (m, 2H), 7.04 (d, *J* = 2.49 Hz, 1H), 7.00 (d, *J* = 7.06 Hz, 1H), 6.96 (dd, *J* = 8.24 and 1.99 Hz, 1H), 6.81 (d, *J* = 8.39 Hz, 1H), 5.98 (d, *J* = 5.39 Hz, 1H), 4.90 (dd, *J* = 5.34 and 12.0 Hz, 1H), 4.38 (dd, *J* = 6.04 and 12.3 Hz, 1H), 4.13~4.06 (m, 2H), 3.87 (s, 3H), 3.84 (s, 3H), 2.04 (s, 3H), 2.02 (s, 3H), 1.45 (t, *J* = 6.89 Hz, 3 H), 1.25 (s, 3H) ppm. ^{13}C NMR (100 MHz, $CDCl_3$) δ = 190.9, 170.8, 169.7, 153.0, 151.0, 148.2, 128.1, 126.0, 120.3, 116.0, 112.4, 111.1, 110.3, 79.1, 74.0, 64.4, 62.7, 56.0, 29.7, 21.1, 20.8, 14.8 ppm.

Synthesis of **VG**

The model compound **VG** was synthesized from 3,4-dimethoxyacetophenone and 2-methoxyphenol, according to the synthetic route reported in the literature [49].

Synthesis of **D_1**

The compound **D_1**, expected to be a product of the Cannizzaro reaction proceeding from **VE_β**, was synthesized in one step using **VE_β** as the starting material, as illustrated in Scheme S3. The synthesis procedure is outlined below.

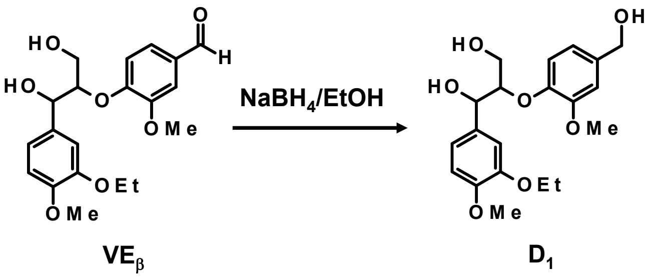

Scheme S3. Synthetic route for D_1.

VE_β (0.05 g, 0.13 mmol) was dissolved in ethanol (5.0 mL) and sodium borohydride (0.010 g, 0.27 mmol) was added, followed by stirring at room temperature. The reaction mixture was reduced in volume by vacuum distillation to about half, and the resulting solution was extracted with dichloromethane. The dichloromethane solution obtained was washed once with brine and dried over anhydrous sodium sulfate. The solvent was removed under reduced pressure to obtain **D_1** (0.043 g, 86.1%) as a colorless oily substance. **D_1** was acetylated with anhydrous acetic acid/pyridine and analyzed by NMR. ^{1}H NMR (400 MHz, $CDCl_3$) δ = 7.0~6.77 (m, 6H), 5.99 (d, *J* = 5.38 Hz, 1H), 5.01 (s, 2H), 4.69~4.63 (m, 1H), 4.41 (dd, *J* = 6.63 and 12.1 Hz, 1H), 4.21 (dd, *J*= 4.48 and 12.1 Hz, 1H), 4.08 (q, *J* = 7.35 Hz, 2H), 3.85 (s, 3H), 3.80 (s, 3H), 2.09 (s, 3H), 2.07 (s,3H), 2.03 (s, 3H), 1.45 (t, *J* = 7.09 Hz, 3H) ppm. ^{13}C NMR (100 MHz, $CDCl_3$) δ = 169.8, 151.0, 149.4, 148.2, 128.8, 127.1, 121.2, 120.0, 118.7, 112.8, 112.2, 111.1, 80.2, 74.0, 66.2, 64.4, 62.8, 56.0, 55.9, 21.1, 20.9, 18.5, 14.8 ppm.

Synthesis of $\mathbf{D_2}$

$\mathbf{D_2}$, the expected product of the Cannizzaro reaction proceeding from $\mathbf{VE_\beta}$, was synthesized in six steps starting from 3,4-dimethoxyacetophenone, as illustrated in Scheme S4. The synthetic procedure is outlined below.

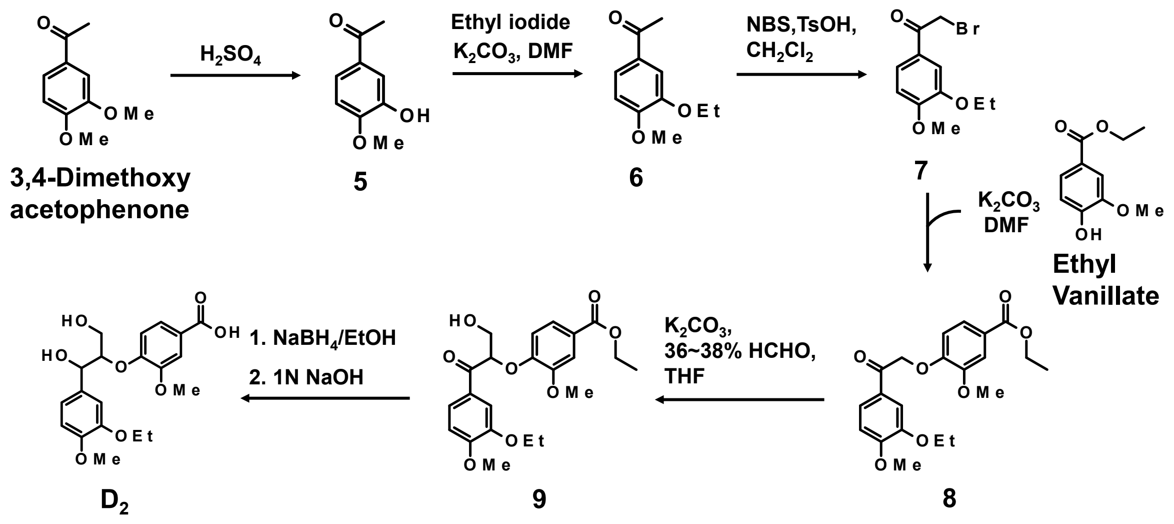

Scheme S4. Synthetic route for $\mathbf{D_2}$.

Synthesis of compound **5**

To a solution of 3,4-dimethoxyacetophenone (3.08 g, 17.1 mmol) in 95% sulfuric acid (15.4 mL, 286.8 mmol), stirred at 65 °C, was added. The reaction progress was monitored by TLC using a 5% iron(III) chloride aqueous solution. After 24 h, the reaction flask was cooled in an ice bath, and 30.0 mL of distilled water was added to dilute the reaction mixture. The resulting mixture was extracted twice with dichloromethane, and the combined organic layers were washed once with brine and dried over anhydrous sodium sulfate. The solvent was removed under reduced pressure, yielding compound **5** (1.1 g, 39.2%) as pale pink crystals. ^{1}H NMR (400 MHz, $CDCl_3$) δ = 7.56~7.53 (m, 2H), 6.89 (d, J = 8.6 Hz, 1H), 5.72 (s, 1H), 3.96 (s, 3H), 2.54 (s, 3H) ppm. ^{13}C NMR (100 MHz, $CDCl_3$) δ = 197.1, 150.6, 145.4, 131.0, 114.5, 109.9, 56.1, 26.5 ppm.

Synthesis of compound **6**

To a solution of compound **5** (0.20 g, 1.2 mmol) and potassium carbonate (0.23 g, 1.6 mmol) in dimethylformamide (1.0 mL) was added ethyl iodide (0.1 mL, 1.3 mmol), and the mixture was stirred at 33 °C. The reaction progress was monitored using a 5% iron(III) chloride aqueous solution. After stirring for 24 h, the reaction flask was cooled to 0-10°C in an ice bath and

stirred for an additional 2 h to quench the reaction. The reaction mixture was then extracted with ethyl acetate, and the organic layer was washed three times with brine and dried over anhydrous sodium sulfate. The solvent was removed under reduced pressure, yielding compound **6** (0.24 g, 104.3%) as white crystals.^{1}H NMR (400 MHz, $CDCl_3$) δ = 7.56 (dd, *J* = 1.95 and 8.38 Hz, 1H), 7.52 (d, *J* = 1.73 Hz, 1H), 6.89 (d, *J* = 8.46 Hz, 1H), 4,16 (q, *J* = 7.01 Hz, 2H), 3.94 (s, 3H), 2.56 (s, 3H), 1.48 (t, *J* = 7.22 Hz, 3H) ppm. ^{13}C NMR (100 MHz, $CDCl_3$) δ = 197.0, 153.5, 148.3, 130.5, 123.2, 111.3, 110.1, 64.4, 56.1, 28.3, 14.7 ppm.

Synthesis of compound **7**

A solution of compound **6** (0.22 g, 1.15 mmol) and *p*-toluenesulfonic acid monohydrate (0.02 g, 0.12 mmol) in dichloromethane was prepared, to which N-bromosuccinimide (0.23 g, 1.27 mmol) was added. The mixture was stirred at 0°C for 10 min, followed by stirring at room temperature. After 24 h, the reaction was quenched by adding 2.0 mL of saturated sodium thiosulfate solution. The reaction mixture was diluted with saturated sodium bicarbonate solution and extracted twice with dichloromethane. The organic layer was washed twice with saturated sodium bicarbonate solution and once with brine, then dried over anhydrous sodium sulfate. After removal of the solvent under reduced pressure, the residue was purified by column chromatography (dichloromethane 100%) to yield compound **7** (0.19 g, 60.9%) as white crystals. ^{1}H NMR (400 MHz, $CDCl_3$) δ = 7.60 (dd, *J* = 2.28 and 8.23 Hz, 1H), 7.53 (d, *J* = 1.98 Hz, 1H), 6.91 (d, *J* = 8.63 Hz, 1H), 4.40 (s, 2H), 4.16 (q, *J*= 7.04 Hz, 2H), 3.95 (s, 3H), 1.49 (t, *J* = 6.76 Hz, 3H) ppm. ^{13}C NMR (100 MHz, $CDCl_3$) δ = 190.2, 154.3, 148.6, 127.0, 123.8, 112.0, 110.3, 64.5, 56.2, 30.5, 14.7 ppm.

Synthesis of compound **8**

A solution of compound **7** (0.19 g, 0.7 mmol), ethyl vanillin (0.16 g, 0.80 mmol), and potassium carbonate (0.11 g, 0.80 mmol) in dimethylformamide (5.0 mL) was prepared. Tetra-n-butylammonium iodide (0.03 g, 0.07 mmol) was added, and the mixture was stirred at room temperature. After stirring for 30 min, the reaction mixture was extracted with ethyl acetate, and the organic layer was washed three times with brine and dried over anhydrous sodium sulfate. The solvent was removed under reduced pressure to afford compound **8** (0.11 g, 39.3%) as white crystals.^{1}H NMR (400 MHz, $CDCl_3$) δ =7.65~7.55 (m, 4H), 6.90 (d, *J* = 8.28 Hz, 1H), 6.77 (d, *J* = 8.16 Hz, 1H), 5.37 (s, 2H), 4.34 (q, *J* = 7.38 Hz, 2H), 3.95 (s, 3H), 3.94 (s, 3H), 1.48 (t, *J* = 7.12 Hz, 3H) ppm. ^{13}C NMR (100 MHz, $CDCl_3$) δ = 192.3, 166.3, 163.4, 151.4, 149.0, 127.5, 124.1, 123.2, 122.6, 112.8, 112.6, 111.5, 110.3, 100.0, 71.3, 64.5, 60.9, 56.2, 56.1, 14.7, 14.4 ppm.

Synthesis of compound **9**

A solution of compound **8** (0.10 g, 0.24 mmol) and potassium carbonate (0.012 g, 0.15 mmol) in tetrahydrofuran (containing BHT) (2.0 mL) was prepared. To this solution, 36-38% formaldehyde aqueous solution (0.008 mL, 0.2 mmol) was added, and the mixture was stirred at 55 °C. After 20 h, the reaction mixture was allowed to return to room temperature, and the reaction was quenched by the addition of acetic acid. The reaction mixture was diluted with saturated sodium hydrogen carbonate solution and extracted once with dichloromethane. The organic layer was washed once with brine and dried over anhydrous sodium sulfate. The solvent was removed under reduced pressure, and the residue was purified by column chromatography (ethyl acetate/hexane = 2/1) to afford compound **9** (0.05 g, 50.0%) as crystals. ^{1}H NMR (400 MHz, $CDCl_3$) δ = 7.72 (dd, *J* = 2.39 and 9.39 Hz, 1H), 7.59 ~7.53 (m, 3H), 6.89 (d, *J* = 8.70 Hz, 1H), 6.79 (d, *J* = 8.19 Hz, 1H), 5.54 (t, *J* = 5.29 Hz, 1H), 4.33 (q, *J* = 7.34 Hz, 2H), 3.94 (s, 3H), 3.91 (s, 3H), 2.93 (t, *J* = 6.66 Hz, 1H), 1.47 (t, *J* = 7.17 Hz, 3H) ppm. ^{13}C NMR (100 MHz, $CDCl_3$) δ = 194.1, 166.1, 154.4, 150.7, 149.4, 127.6, 124.9, 123.4, 115.2, 113.0, 112.0, 110.4, 83.3, 64.5, 63.8, 61.0, 56.2, 56.0, 14.7, 14.4 ppm.

Synthesis of $\mathbf{D_2}$

A solution of compound **9** (0.02 g, 0.05 mmol) in dichloromethane was prepared, and sodium borohydride (0.004 g, 0.1 mmol) was added. The mixture was stirred at room temperature. After 1.5 h, the reaction was quenched by the addition of distilled water, and the reaction mixture was extracted with dichloromethane. The organic layer was washed once with brine and dried over anhydrous sodium sulfate. The solvent was removed under reduced pressure. The resulting residue was dissolved in ethanol, and 1N aqueous sodium hydroxide solution (0.48 mL, 0.48 mmol) was added. The mixture was stirred at room temperature. After 2 h, the reaction mixture was extracted with dichloromethane, and the organic layer was washed once with brine and dried over anhydrous sodium sulfate. The solvent was removed under reduced pressure to afford $\mathbf{D_2}$ (0.02 g, ~100%) as a mixture of diastereomers. ^{1}H NMR (400 MHz, $CDCl_3$) δ =7.71~7.57 (m, 2H), 7.00~6.90 (m, 3H), 6.84 (dd, *J* = 5.02 and 8.30 Hz, 1H), 4.99 (t, *J* = 3.82 Hz, 1H), 4.29~4.24 (m, 1H), 4.13~4.05 (m, 2H), 3,92 (d, *J* = 15.3 Hz, 3H), 3.69 (dd, *J* = 3.4 and 12.0 Hz, 1H), 3.59 (dd, *J* = 4.49 and 12.2 Hz, 1H), 2.11 (s, 3H), 1.45 (t, *J* = 7.3 Hz, 3H) ppm. ^{13}C NMR (100 MHz, $CDCl_3$) δ = 205.8, 177.0, 170.9, 152.6, 149.4, 148.5, 131.7, 124.6, 119.5, 118.3, 113.3, 111.4, 111.3, 73.9, 73.2, 64.4, 56.1, 56.0, 20.8, 14.8 ppm.
